# Mixing I and Br in Inorganic Perovskites: Atomistic Insights from Reactive Molecular Dynamics Simulations

**DOI:** 10.1021/acs.jpcc.4c00563

**Published:** 2024-02-23

**Authors:** Mike Pols, Adri C. T. van Duin, Sofía Calero, Shuxia Tao

**Affiliations:** † Materials Simulation & Modelling, Department of Applied Physics and Science Education, Eindhoven University of Technology, 5600 MB Eindhoven, The Netherlands; ‡ Center for Computational Energy Research, Department of Applied Physics and Science Education, Eindhoven University of Technology, 5600 MB Eindhoven, The Netherlands; § Department of Mechanical Engineering, Pennsylvania State University, University Park, Pennsylvania 16802, United States

## Abstract

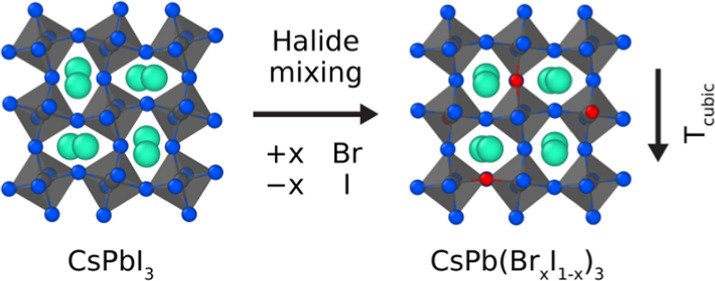

All-inorganic halide perovskites have received a great deal of attention as attractive alternatives to overcome the stability issues of hybrid halide perovskites that are commonly associated with organic cations. To find a compromise between the optoelectronic properties of CsPbI_3_ and CsPbBr_3_, perovskites with CsPb(Br_*x*_I_1–*x*_)_3_ mixed compositions are commonly used. An additional benefit is that without sacrificing the optoelectronic properties for applications such as solar cells or light-emitting diodes, small amounts of Br in CsPbI_3_ can prevent the inorganic perovskite from degrading to a photo-inactive non-perovskite yellow phase. Despite indications that strain in the perovskite lattice plays a role in the stabilization of the material, a full understanding of such strain is lacking. Here, we develop a reactive force field (ReaxFF) for perovskites starting from our previous work for CsPbI_3_, and we extend this force field to CsPbBr_3_ and mixed CsPb(Br_*x*_I_1–*x*_)_3_ compounds. This force field is used in large-scale molecular dynamics simulations to study perovskite phase transitions and the internal ion dynamics associated with the phase transitions. We find that an increase of the Br content lowers the temperature at which the perovskite reaches a cubic structure. Specifically, by substituting Br for I, the smaller ionic radius of Br induces a strain in the lattice that changes the internal dynamics of the octahedra. Importantly, this effect propagates through the perovskite lattice ranging up to distances of 2 nm, explaining why small concentrations of Br in CsPb(Br_*x*_I_1–*x*_)_3_ (*x* ≤ 1/4) have a significant impact on the phase stability of mixed halide perovskites.

## Introduction

Halide perovskites hold a great promise for a variety of optoelectronic applications, which include photovoltaics, light-emitting diodes (LEDs), and photodetectors.^1−4^ The main appeal of halide perovskites stems from the combination of facile synthesis methods and a highly tunable AMX_3_ perovskite crystal lattice.^5,6^ By changing or mixing the A-site cation (MA^+^, FA^+^, Cs^+^), M-site metal cation (Pb^2+^, Sn^2+^), and X-site halide anion (I^–^, Br^–^, Cl^–^), a large compositional space with varying material properties can be explored for specific applications.^7,8^ Despite these beneficial material characteristics, the commercialization of perovskite optoelectronic devices has thus far been hampered by long-term stability issues.^9,10^ Several of these stability issues, such as a poor thermal stability and material decomposition upon contact with water, can be attributed to the volatile and hydrophilic nature of commonly incorporated organic A-site cations (MA^+^ and FA^+^).^11−13^

One strategy that has been proposed to overcome the stability issues related to organic cations is the use of all-inorganic halide perovskites. Such all-inorganic halide perovskites, in which Cs^+^ is the sole A-site cation, have shown to be more resistant to external stimuli such as thermal stress and moisture.^14^ As a result of this, they have been used in a variety of applications. For example, CsPbI_3_, with its relatively low band gap (1.73 eV^15^), is ideal for solar cells^16^ and LEDs emitting red light,^17^ whereas CsPbBr_3_, with its larger band gap (2.37 eV^18^), is commonly used in tandem solar cells,^19^ green LEDs,^20^ and photodetectors.^21^ Moreover, such all-inorganic perovskites can be tuned through nanostructuring, offering improved stability and optoelectronic properties for an even wider range of applications.^22^

Nevertheless, all-inorganic perovskites are not without any problems, as indicated by the poor phase stability of CsPbI_3_. It is well established that CsPbI_3_ transforms from a cubic (α) to tetragonal (β) to orthorhombic (γ) phase, going from high to progressively lower temperatures.^23,24^ Due to a mismatch of the ionic radii in the lattice, evidenced by the low Goldschmidt tolerance factor of CsPbI_3_ (0.807),^25,26^ the low-temperature γ-phase is rather distorted, making it prone to convert into a non-perovskite yellow (δ) phase.^23,24,27^ The yellow phase of CsPbI_3_ is photoinactive, which is ill-suited for optoelectronic applications. On the contrary, resulting from the better fit of the ions in the lattice indicated by its higher Goldschmidt tolerance factor (0.815),^25,26^ CsPbBr_3_ does not show any degradation to a yellow phase. Therefore, I^–^ and Br^–^ ions are commonly mixed to increase the phase stability of all-inorganic perovskites. A variety of works have demonstrated, that apart from impacting the optoelectronic properties,^28−30^ the mixing of Br into a CsPbI_3_ enhances the stability of CsPb(Br_*x*_I_1–*x*_)_3_ films, either slowing down or preventing the yellow phase from forming altogether.^29−34^ Näsström et al.^35^ systematically studied the phase transitions of CsPb(Br_*x*_I_1–*x*_)_3_ perovskites, from which they found that a gradual increase of the Br content in mixed halide perovskites lowers the temperatures at which the perovskite transforms into the cubic phase. Although lattice strain has been proposed as the mechanism responsible for α-phase stabilization,^15,32^ the atomistic effects of halide mixing on the various perovskite phases remain unclear.

Recently, using reactive force field (ReaxFF) molecular dynamics simulations, we studied the phase transitions and degradation reactions at surfaces and grain boundaries of CsPbI_3_.^36,37^ In this work, we extend our study to the lattice and ion dynamics of mixed halide perovskites. Starting from our ReaxFF parameter set for CsPbI_3_,^36^ we expand the force field to CsPbBr_3_ and mixed CsPb(Br_*x*_I_1–*x*_)_3_ perovskites. After validating the new ReaxFF parameters on the equations of state, mixing enthalpies, degradation reactions, and defect migration barriers, we apply our model in large-scale molecular dynamics simulations of mixed perovskites. By combining information from the phase diagrams with the microscopic order in the octahedral orientations, we provide important atomistic insights into the effects of halide mixing.

## Methods

We train the ReaxFF parameters for CsPb(Br_*x*_I_1–*x*_)_3_ halide perovskites against reference data from density functional theory (DFT) calculations performed in VASP^38−40^ and ADF^41,42^ using the PBE + D3(BJ)^43,44^ exchange–correlation functional. The reference data includes atomic charges, equations of state of different perovskite and non-perovskite phases, equations of state of precursors (e.g., CsX and PbX_2_ with X = I/Br), defect formation energies, defect migration barriers, and phase transitions of compounds. Full details of these calculations are found in Supporting Information Note 1. The agreement between the reference data and the predictions from the ReaxFF parameter set {*p*_*j*_} is captured by a sum of squared errors (SSE) loss function as
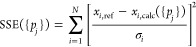
1where *x*_*i*,ref_ and *x*_*i*,calc_ are the reference values and ReaxFF predictions for an entry *i* in the training set and σ_*i*_ the weight of that entry. The final ReaxFF parameter set is obtained by minimizing the SSE loss function using the covariance matrix adaptation evolution strategy,^45^ as implemented in ParAMS in AMS2022.^46,47^ We use the previously published I/Pb/Cs parameters^36^ for inorganic halide perovskites as the initial point for parameter optimization. Without any ReaxFF parameters for Br available in the literature, the starting point for the Br parameters was obtained by scaling the interactions of I with other species. Details of the parameter optimization procedure and the scaling of the interatomic interactions can be found in Supporting Information Note 2.

## Results and Discussion

### Force Field Validation

**Figure 1:**
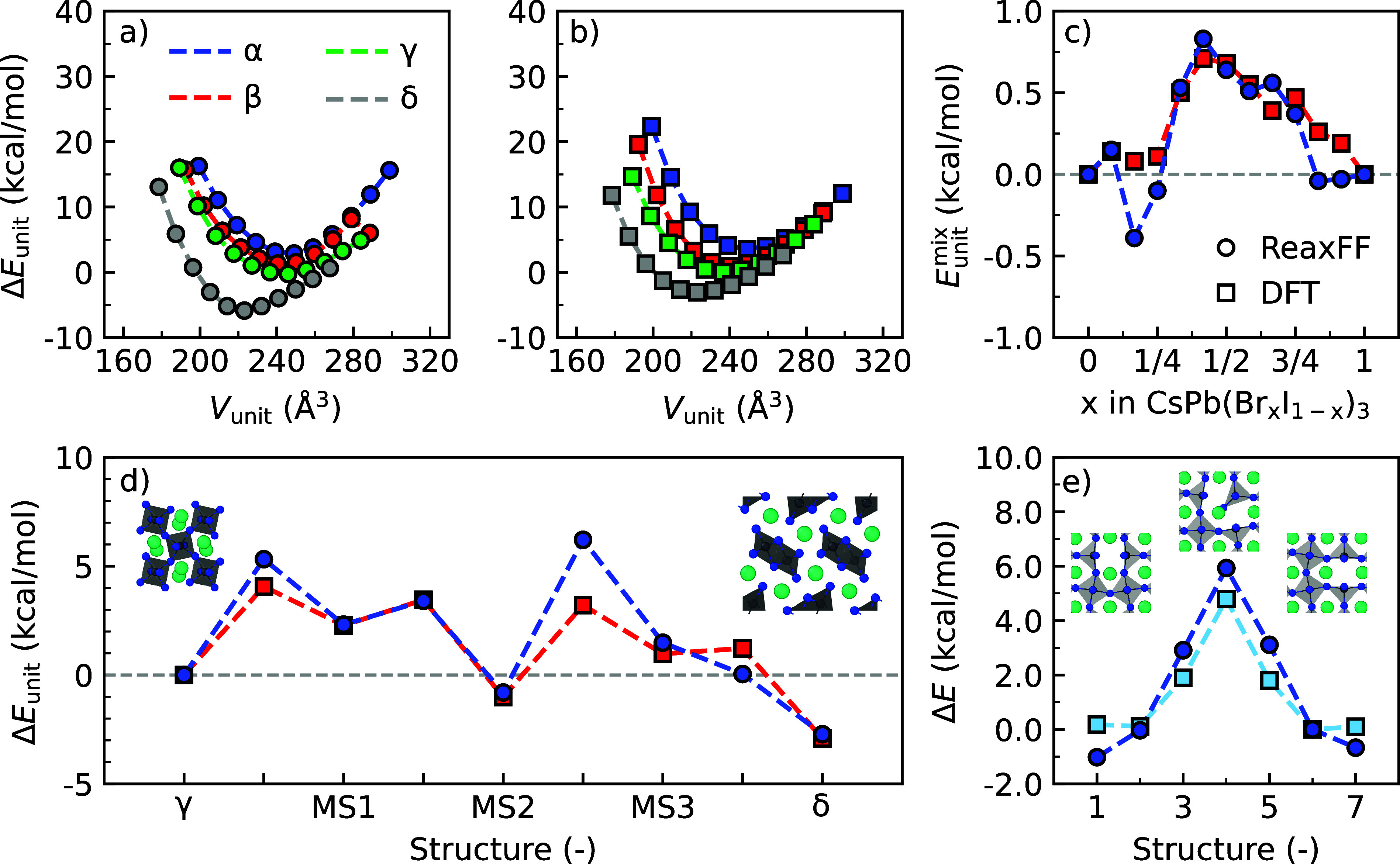
Equations of state of various perovskite and non-perovskite phases of CsPbI_3_ from (a) ReaxFF and (b) DFT calculations. (c) Mixing enthalpies of CsPb(Br_*x*_I_1–*x*_)_3_ perovskites. (d) CsPbI_3_ degradation mechanism from the γ-phase to the δ-phase. (e) Defect migration barrier of I vacancy in CsPbI_3_. Data from ReaxFF simulations and DFT calculations are shown in circles and squares, respectively. Degradation mechanism reproduced with permission from ref (48). Copyright 2022 Elsevier.

Using the above-mentioned optimization procedure, we obtain a ReaxFF description for the elements I/Br/Pb/Cs that exhibit good agreement with the DFT reference data in the training set, as shown in Figure 1 and Supporting Information Note 3. The obtained ReaxFF parameter set is provided in the Supporting Information. Focusing on pure compounds first, we note that the equations of state of the various phases of CsPbI_3_, both perovskite (α-, β-, and γ-phase) and non-perovskite (δ-phase), as obtained with ReaxFF (Figure 1a), are in good agreement with DFT calculations (Figure 1b). We find that the ReaxFF parameter set correctly ranks the total energies of the various bulk phases of CsPbI_3_ from least to most stable as α < β < γ < δ. Moreover, in agreement with the reference data, the ReaxFF parameter set predicts a similar stability trend for the different phases of CsPbBr_3_, an overview of which is shown in Figure S1 and Table S5. As shown in Figure 1c, the ReaxFF force field also predicts positive mixing enthalpies for mixed halide compositions (<1.0 kcal/mol per formula unit), in agreement with DFT calculations. We hypothesize that the discrepancies in the mixing enthalpies at *x* = 1/6 and *x* = 1/4 can be linked to overstabilized mixed perovskite structures, more details of which are provided in Supporting Information Note 3.

Shifting our focus from pristine bulk systems to degradation reactions and defective perovskites, we find that such systems are represented well by the new ReaxFF parameter set (Figure 1d). In particular, the new ReaxFF force field captures the energetics of the degradation of CsPbI_3_ from the orthorhombic (γ) to the yellow (δ) phase, as predicted by DFT calculations for the structures from Chen et al.^48^ Compared to the previously published I/Pb/Cs parameter set (Figure S2),^36^ the reparameterized force field provides considerable improvements for the stability of the metastable states (MS1, MS2, and MS3) and final state (δ) in the degradation pathway, potentially paving the way for the simulation of this degradation reaction using rare event sampling methods. Finally, we find that the ReaxFF force field finds defect migration barriers of halide point defects (i.e., vacancies and interstitials) that are in line with migration barriers from DFT calculations. Figure 1e demonstrates that the migration of an I vacancy in CsPbI_3_ from ReaxFF 4.8 kcal/mol is close to that from DFT calculations 7.0 kcal/mol. Defect migration barriers of other types of defects, such as an I interstitial in CsPbI_3_ or a Br vacancy or interstitial in CsPbBr_3_, are also correctly predicted by the new ReaxFF force field, and an overview of these barriers is shown in Figure S3.

**Figure 2:**
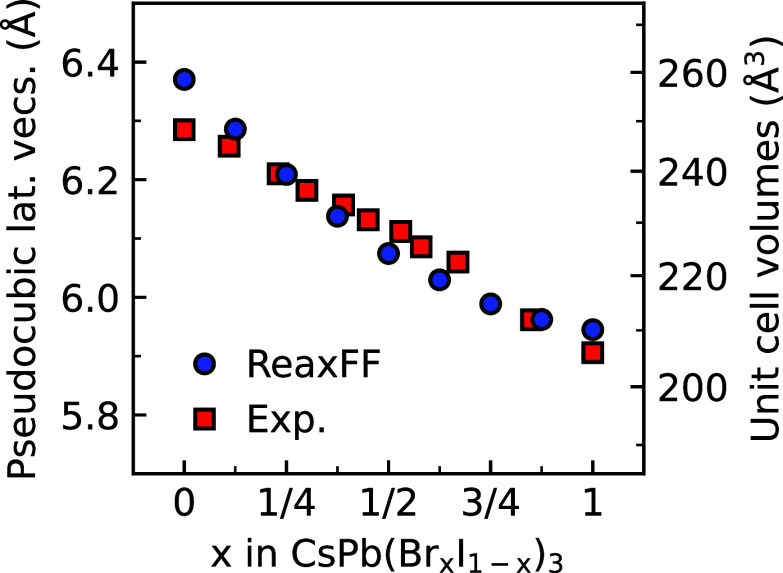
Pseudocubic lattice vectors and unit cell volumes of CsPb(Br_*x*_I_1–*x*_)_3_ perovskites. Comparison of experimental data (squares) with ReaxFF simulations (circles) at 575 K. Experimental data from ref (35).

To assess the performance of the new parameter set during finite temperature simulations, we compare unit cell volumes from simulations with experimentally observed volumes^35^ in Figure 2. The full details of the creation of the model systems and the simulations can be found in Supporting Information Notes 4 and 5. Notably, we find that the ReaxFF simulations predict volumes within 1% of experiments, demonstrating that an increase in the Br content in the CsPb(Br_*x*_I_1–*x*_)_3_ lattice decreases the unit cell volumes. An effect that can be attributed to the smaller ionic radius of Br (1.96 Å) compared to that of I (2.20 Å),^26^ which reduces the size of the crystal lattice.

### Phase Diagrams

**Figure 3:**
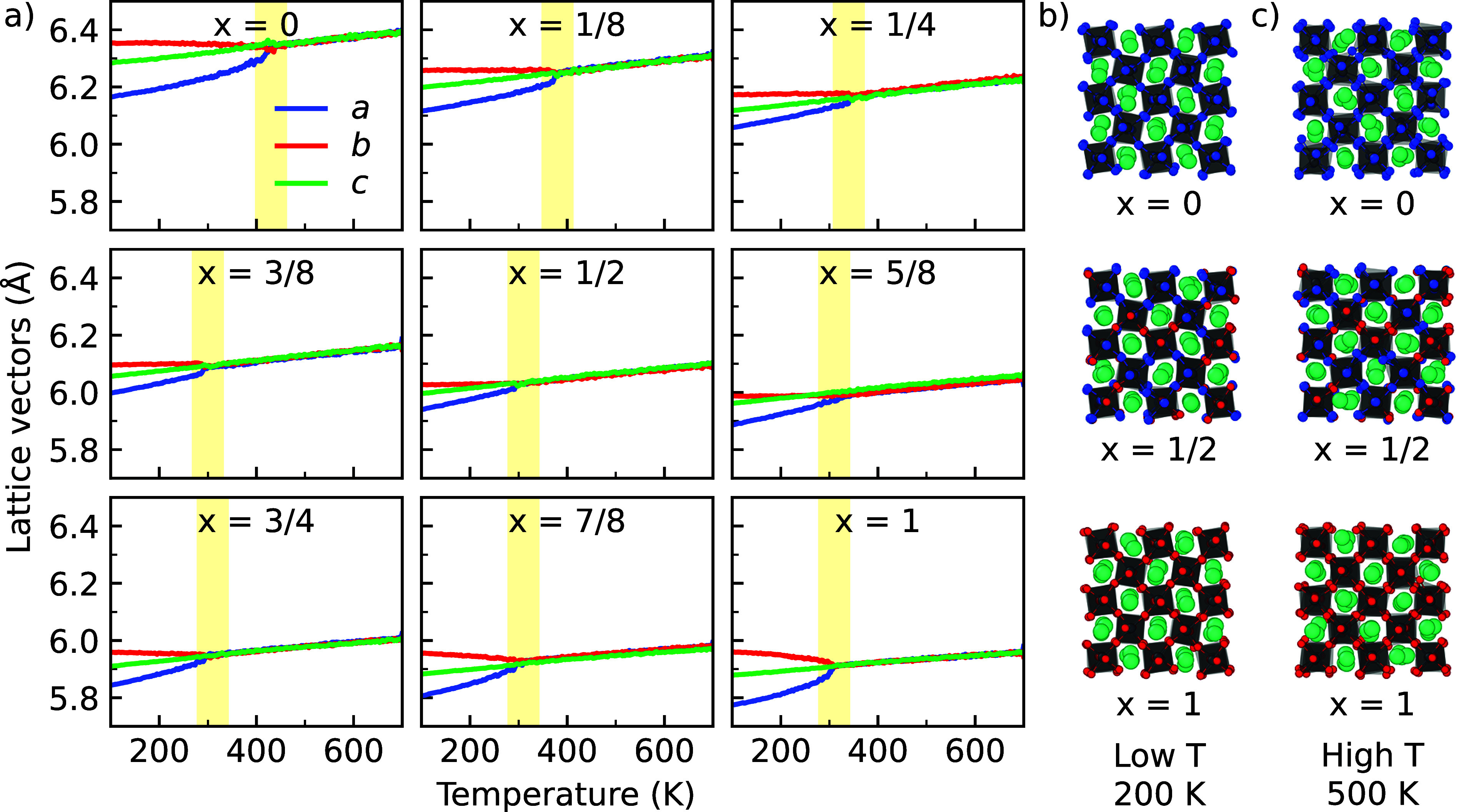
(a) Phase diagrams of CsPb(Br_*x*_I_1–*x*_)_3_ perovskites with varying compositions obtained during the gradual heating of the inorganic compounds. Snapshots of mixed halide perovskites with *x* = 0, *x* = 1/2, and *x* = 1 compositions are shown in (b) 200 K and (c) 500 K. The yellow bars indicate the temperature at which the cubic phase is initially observed. The pseudocubic lattice vectors *a*, *b*, and *c* are shown in all figures.

Having established that the ReaxFF parameter set can appropriately describe the macroscopic properties of mixed compositions at various temperatures, we now shift our focus to studying the transitions among the various perovskite phases. To do so, we gradually heat different CsPb(Br_*x*_I_1–*x*_)_3_ systems with varying amounts of Br (*x* = 0, 1/8, 1/4, 3/8, 1/2, 5/8, 3/4, 7/8, and 1) from 100 to 700 K and monitor the temperature evolution of the lattice vectors in Figure 3. Details of the simulations used to obtain the phase diagrams can be found in Supporting Information Note 5.

Looking into the phase diagrams of the pure perovskites (*x* = 0 and 1 in Figure 3a), we observe that both perovskites transition from the low-temperature orthorhombic phase to a high-temperature cubic phase. As shown in the snapshots in Figures 3b,c, the perovskites change from a phase in which the octahedra are arranged in an ordered tilted fashion at low temperatures (200 K), to one where this order in the octahedral tilting is overcome by the dynamic alternation between many different tilts at high temperatures (500 K). We distinguish these phases based on the magnitude of the lattice vectors; in the orthorhombic phase all lattice vectors are different (*a* ≠ *b* ≠ *c*) and in the cubic phase all vectors are the same length (*a* = *b* = *c*). The intermediate tetragonal phase (*a* = *b* ≠ *c*) only appears during a narrow temperature window for pure CsPbI_3_ in Figure 3 (410 to 430 K), as a result of rapid thermal fluctuations. In agreement with experiments,^21,24,35^ we find that CsPbBr_3_ (310 K) transforms to the cubic phase at lower temperatures compared to CsPbI_3_ (430 K). This difference in the phase transition temperatures indicates that a smaller amount of thermal fluctuations is needed for phase transitions to occur in CsPbBr_3_,^49,50^ an observation that can be linked to the aforementioned higher Goldschmidt tolerance factor of CsPbBr_3_ (0.815) compared to that of CsPbI_3_ (0.807).^25,26^ It should be noted that the phase transition temperatures from ReaxFF underestimate the experimental phase transition temperatures by approximately 50 to 100 K for both CsPbBr_3_ and CsPbI_3_. We relate this overprediction to the exchange–correlation functional used for the training set (i.e., PBE + D3(BJ)), the choice of which has an impact on the phase transition temperatures.^51^

Focusing on the mixed compositions, we find that the mixing of Br into CsPbI_3_ significantly lowers the phase transition temperature to that of the cubic phase of perovskites. Furthermore, the phase diagrams in Figure 3a show that the largest part of the drop in the phase transition temperature occurs at relatively low Br concentrations (*x* ≤ 1/4), leveling off for concentrations from *x* = 1/4 onward. This finding is consistent with earlier experimental investigations in which it was also found that the phase transition temperature of mixed halide perovskites depends nonlinearly on the Br concentration, with the largest drop occurring for small amounts of Br.^35,52^

### Octahedral Dynamics

**Figure 4:**
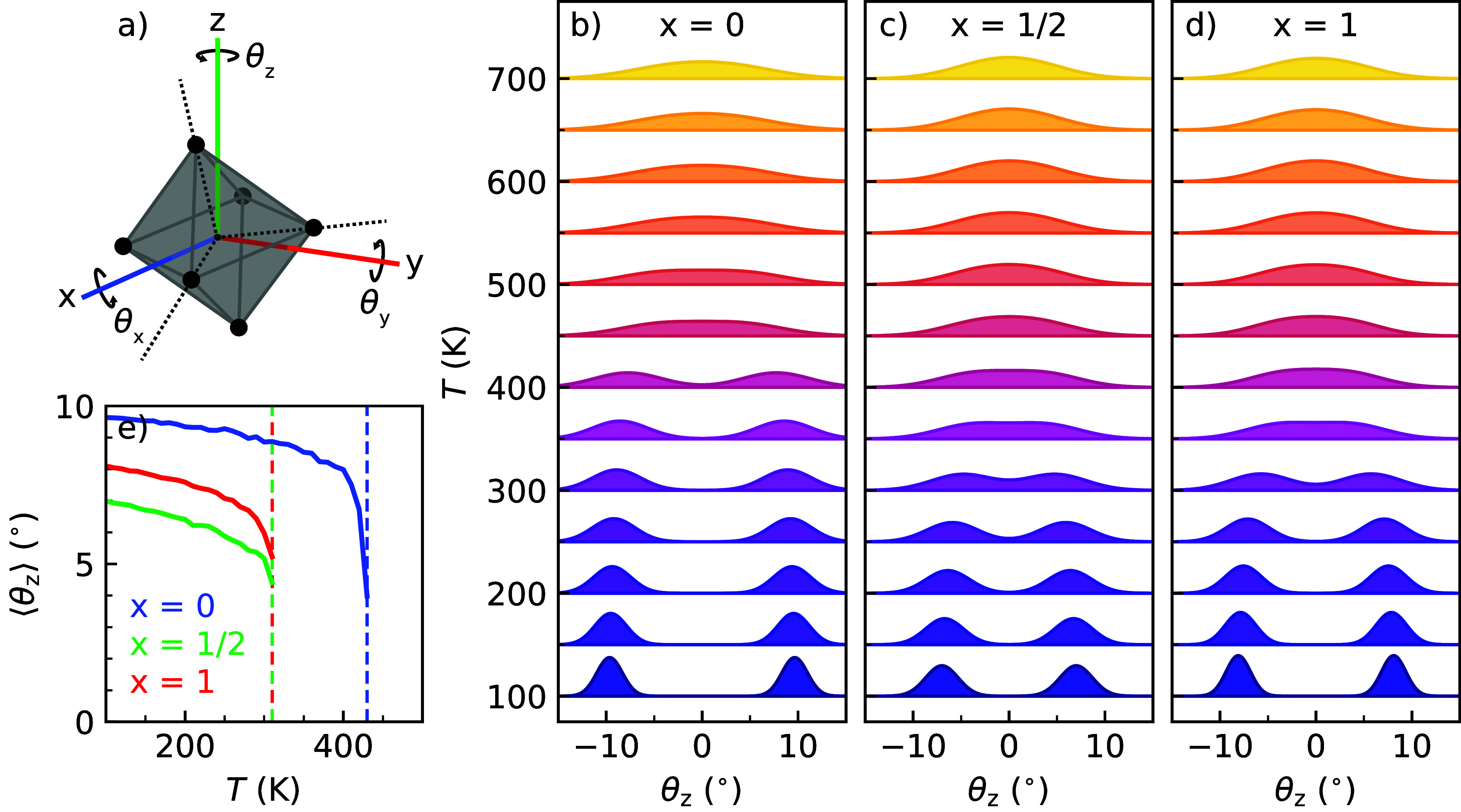
(a) Angles θ_*x*_, θ_*y*_, and θ_*z*_ used to determine the orientation of the PbX_6_ octahedra. Temperature evolution of the octahedral orientation θ_*z*_ for CsPb(Br_*x*_I_1–*x*_)_3_ perovskites with compositions (b) *x* = 0, (c) *x* = 1/2, and (d) *x* = 1. (e) Temperature evolution of the average tilting angle ⟨θ_*z*_⟩ for different mixed halide perovskite compositions.

To gain more insight into the phase behavior of CsPb(Br_*x*_I_1–*x*_)_3_, we analyze the orientation of the PbX_6_ octahedra in the lattice. Using the method outlined by Wiktor et al.,^53^ the orientation of the octahedra with respect to the cubic lattice can be described by the angles θ_*x*_, θ_*y*_, and θ_*z*_, following the convention shown in Figure 4a. The angles act as a measure of the degree with which the octahedra are distorted in the perovskite lattice. We obtain insights into the effects of halide mixing on the internal dynamics by examining the temperature progression of the octahedral tilting from continuously heated runs at atmospheric pressure for various compositions. The full simulation details and the procedure used to extract the octahedral orientation from the simulations are found in Supporting Information Notes 5 and 6.

The temperature evolution of θ_*z*_ is shown in Figure 4b–d for various compositions, whereas the evolution of θ_*x*_ and θ_*y*_ can be found in Figures S10 and S11. All angles, θ_*x*_, θ_*y,*_ and θ_*z*_, change from a bimodal distribution around zero at low temperatures to a single broad distribution centered at zero at high temperatures. This indicates that all compositions progress from a low-temperature phase in which the octahedra have a regularly distorted arrangement to a high-temperature phase that lacks any instantaneous order but on average has a non-distorted tilting pattern. We note that these observations are in line with the phase transitions of perovskites where the material progresses from an orthorhombic phase into a cubic phase upon gradual heating, as shown in Figure 3. Further analysis of the tilting distributions, by means of a symmetric double Gaussian fit (Figure 4e), allows for the comparison of the various compositions at temperatures ranging from 100 to 430 K. The comparison illustrates that PbX_6_ octahedra in CsPbI_3_ have an on average larger tilt than those in CsPbBr_3_. Interestingly, we find that the mixed halide perovskite (*x* = 1/2) exhibits a smaller average tilt angle and wider tilt distributions for all angles than either of the pure compounds (*x* = 0 or *x* = 1), which can be linked to substantial atomistic changes in the perovskite lattice as a result of the halide mixing.

### Atomistic Effects of Halide Mixing

**Figure 5:**
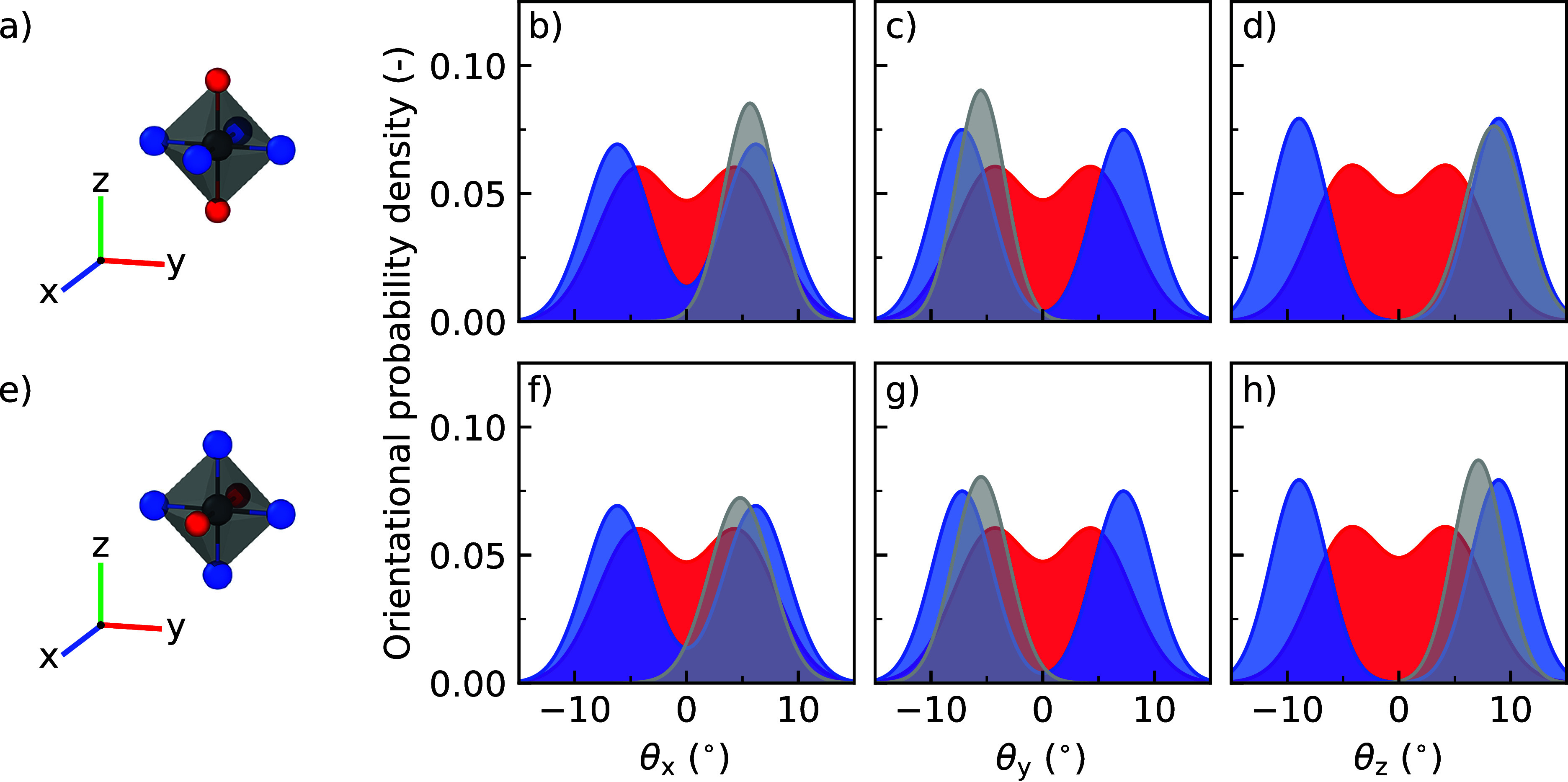
Tilting distributions of PbX_6_ octahedra. (a) Two Br substitutions at the axial position with (b–d) showing the distributions of θ_*x*_, θ_*y*_, and θ_*z*_. (e) Two Br substitutions at the equatorial position with (f–h) showing the distributions of θ_*x*_, θ_*y*_, and θ_*z*_. The tilting distributions of the substituted octahedra are shown in gray and those for the pure compounds CsPbI_3_ and CsPbBr_3_ are shown in blue and red, respectively.

Finally, to explore the atomistic effects of halide mixing, we analyze the tilting distributions of the PbX_6_ octahedra in the dilute limit. By mixing small amounts of Br into pure CsPbI_3_, we can identify the atomistic effects that such substitutions have on the octahedral tilting. In Figure 5 we focus on the tilting distributions of Br-substituted PbI_6_ octahedra and compare them with the tilting distributions of octahedra in pure CsPbI_3_ and CsPbBr_3_. To prevent thermal motion from dominating the motion of the octahedra in CsPbI_3_, we investigate the mentioned effects in the low-temperature γ-phase at 300 K. In this phase, two types of halide substitutions are possible: (1) axial halide substitutions along the *z*-direction of the octahedra (Figure 5a) and (2) equatorial substitutions in the *xy*-plane of the octahedra (Figure 5e). Both types of substitutions are investigated. An overview of the simulation details and model systems used during the simulations can be found in Supporting Information Note 5, whereas additional analyses of the octahedral tilting are found in Supporting Information Note 7.

The tilting distributions in Figure 5 show that halide substitutions impact the orientation of octahedra. For both types of substitutions, axial (Figure 5b–d) and equatorial (Figure 5f–h), the orientation of the Br-substituted PbI_6_ octahedra shifts away from that of pure CsPbI_3_ to that of CsPbBr_3_ by decreasing by about 1 to 2°. Besides, the octahedral tilting distributions become more narrow. The exact values of the shift and narrowing of the tilting distributions can be found in Table S9. Together, the decreasing tilt angles and the narrowing of the tilting distributions indicate a restrained motion for the substituted octahedra. Specifically, whenever Br connects two octahedra in a perovskite lattice that predominantly consists of PbI_6_ octahedra, a strained interconnect is formed between the substituted octahedra as a result of the previously mentioned smaller size of Br compared to that of I, which leads to shorter bond lengths. To alleviate this strain, the substituted octahedra adjust themselves to an overall less tilted geometry, which closely resembles the cubic phase, at low temperatures. This effect is largest for octahedral orientations perpendicular to the substitution direction, for example, θ_*x*_ and θ_*y*_ for axial substitutions. Although the effect of substituting two Br into one PbX_6_ octahedron is demonstrated here, we note that the substitution of a single Br into an octahedron has similar effects as shown in Figure S14.

**Figure 6:**
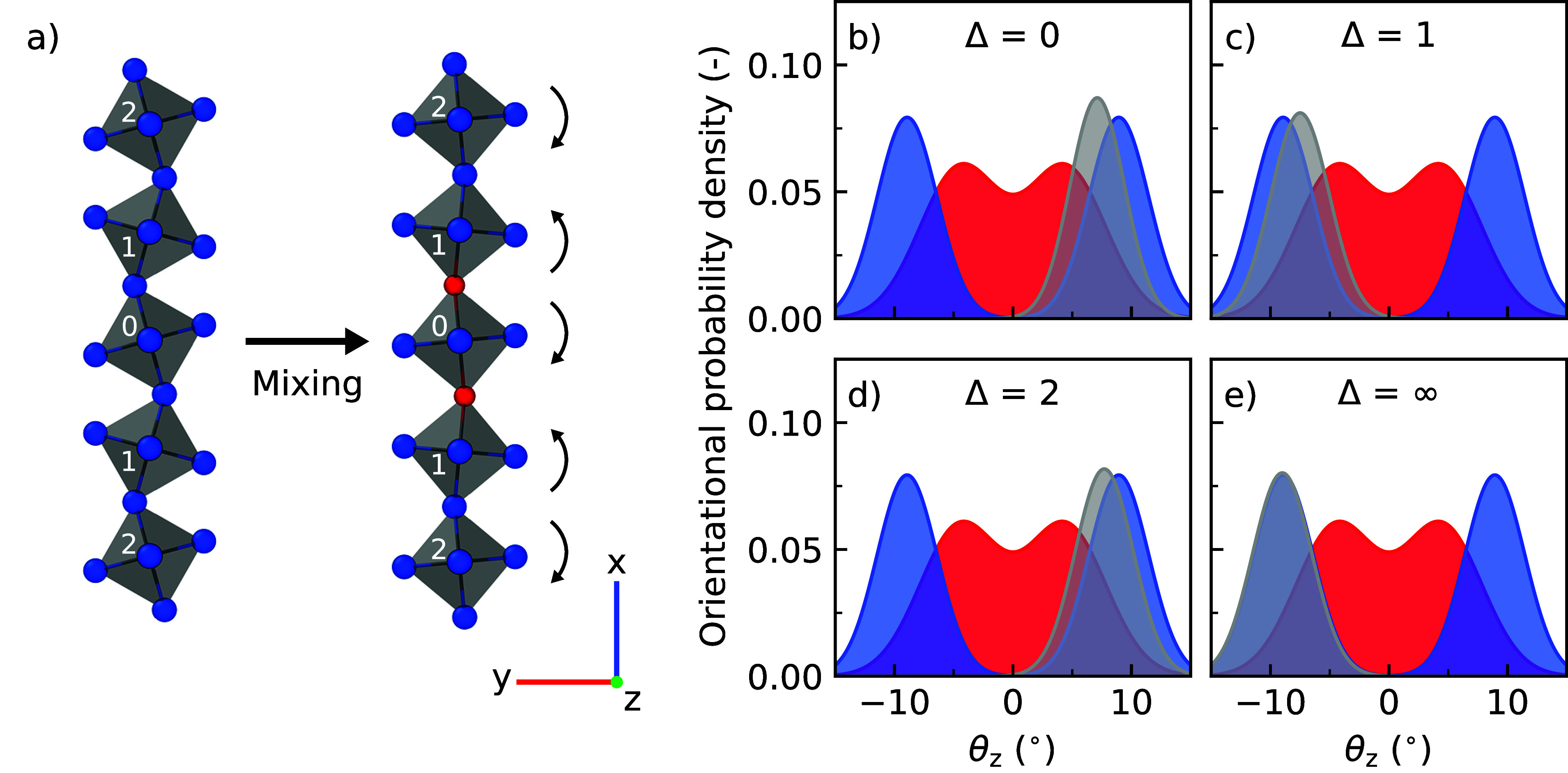
(a) Non-substituted and double Br-substituted chains of PbX_6_ octahedra. The numbers in the octahedra indicate the distance relative to the substituted octahedron. Distribution of θ_*z*_ of (b) substituted octahedron (Δ = 0), (c) direct neighbor of the substituted octahedron (Δ = 1), (d) octahedron two sites away from the substituted octahedron (Δ = 2), and (e) reference octahedron very far away from the halide substitution (Δ = ∞). The tilting distributions of the investigated octahedra are shown in gray, and those for CsPbI_3_ and CsPbBr_3_ in blue and red, respectively.

To investigate the range of the effect of halide substitutions, we monitor the octahedral tilting of octahedra close to an octahedron with two equatorial substitutions, as shown in Figure 6. A schematic overview of the octahedra that were considered is shown in Figure 6a. We find that the tilting distributions of the octahedra close to the substitution (Figure 6b–d) deviate from the tilting distributions observed in pure CsPbI_3_. The affected octahedra show a smaller average angle and a more narrow distribution for θ_*z*_ as shown in Table S10. The effect diminishes for octahedra far away from the halide substitutions (Figure 6e), becoming negligible for octahedra spaced further than three sites away from the substitution (Δ > 3) as shown in Figure S15 and Table S11. We identified the propagation distance of this effect to be about 2 nm. Interestingly, this propagation is not only found in the direction of the halide substitution as shown in Figure 6 but also in directions perpendicular to the substitutions, albeit at a shorter range (<1 nm) as seen in Figure S16 and Table S12. As a consequence of the propagation of this effect, small concentrations of halide substitutions can have profound effects on the octahedral dynamics of perovskites. These atomistic insights are important for understanding why low levels of Br (*x* ≤ 1/4) are sufficient to stabilize the cubic phase in CsPb(Br_*x*_I_1–*x*_)_3_ perovskites.

## Conclusions

In summary, we developed a I/Br/Pb/Cs ReaxFF parameter set for inorganic halide perovskites. We demonstrate that the developed force field is suitable for describing the various perovskite and non-perovskite phases of pure CsPbI_3_, pure CsPbBr_3_, and mixed CsPb(Br_*x*_I_1–*x*_)_3_ compounds. By studying the phase transitions of CsPb(Br_*x*_I_1–*x*_)_3_ perovskites, we find that progressively increasing the Br content stabilizes the high-temperature cubic phase. We highlight that a large part of the stabilization effect comes from the initial Br substitutions (*x* ≤ 1/4). An investigation of the octahedral tilting distributions in mixed perovskites shows that halide mixing induces strain in the lattice, causing the perovskite to adopt a more cubic structure. Importantly, the effect of this strain propagates to octahedra close to the substitution, reaching distances of up to 2 nm. These results provide fundamental insights into the microscopic effects of strain that result from halide mixing and are valuable in the development of optoelectronic devices based on inorganic halide perovskites. Finally, we expect the newly developed ReaxFF parameters to also be used to study other important phenomena, such as defect migration and degradation reactions, occurring in inorganic mixed halide perovskites of various dimensions (e.g., 2D and quantum dots) with large-scale molecular dynamics simulations.

## Supporting Information Available

List of I/Br/Pb/Cs ReaxFF parameters that were obtained from the parameter optimization procedure (TXT)
Training set used to optimize the I/Br/Pb/Cs ReaxFF parameters, including the job collection file with all structures, the data set file with the energies and charges, and an overview of the performance of the final ReaxFF force field for the entries in the training set (ZIP)
Computational settings of the training set entries; parameter optimization procedure; ReaxFF force field validation tests; method used for creation of mixed halide perovskites; molecular dynamics simulation details; methods for extraction and analysis of octahedral tilting in perovskites; analysis of the strain effect and its propagation through the perovskite lattice; and tolerance factors of inorganic halide perovskites (PDF)
