# Supplementary material for: Mixing I and Br in Inorganic Perovskites: Atomistic Insights from Reactive Molecular Dynamics Simulations
Source: J Phys Chem C Nanomater Interfaces. 2024 Feb 23;128(9):4111–8. doi: 10.1021/acs.jpcc.4c00563 (PMC10926166; doi:10.1021/acs.jpcc.4c00563)
Supplement: Supplementary file 3 — jp4c00563_si_003.pdf [file jp4c00563_si_003.pdf]

Supporting Information: Mixing I and Br in Inorganic Perovskites: Atomistic Insights from Reactive Molecular Dynamics Simulations

Mike Pols,[†,‡] Adri C.T. van Duin,[¶] Sofía Calero,[*,†] and Shuxia Tao[*,†,‡]

†*Materials Simulation & Modelling, Department of Applied Physics and Science Education, Eindhoven University of Technology, 5600 MB, Eindhoven, The Netherlands*

‡*Center for Computational Energy Research, Department of Applied Physics and Science Education, Eindhoven University of Technology, 5600 MB, Eindhoven, The Netherlands*

¶*Department of Mechanical Engineering, Pennsylvania State University, University Park, PA 16802, United States*

E-mail: s.calero@tue.nl; s.x.tao@tue.nl

Contents

Supporting Notes **S4**

1 Reference data . . . S4

1.1 Periodic systems . . . S4

1.2 Molecular systems . . . S6

2 Force field optimization . . . S7

2.1 Parameter optimization . . . S7

2.2 Parameter scaling . . . S7

3 Force field validation . . . S9

3.1 Atomic charges . . . S9

3.2 Equations of state . . . S10

3.3 Mixing enthalpies . . . S11

3.4 $CsPbI_3$ degradation pathway . . . S12

3.5 Defect migration barriers . . . S14

3.6 PbX_2 molecular geometries . . . S15

3.7 PbI_2 precursor geometry . . . S16

4 Mixed halide model systems . . . S17

5 Molecular dynamics simulations . . . S18

5.1 Unit cell volumes . . . S18

5.2 Phase diagrams . . . S19

5.3 Homogeneously mixed systems . . . S21

5.4 Dilute mixed systems . . . S23

6 Octahedral orientation . . . S24

6.1 Determination of orientation . . . S24

6.2 Analysis of orientation . . . S25

7 Substitution propagation . . . S27

7.1 Single substitution . . . S27

7.2 Propagation distance S28
7.3 Perpendicular propagation S29
8 Tolerance factors S31
References **S32**

Supporting Notes

1 Reference data

The reference data used in the training set was generated using density functional theory (DFT) calculations. Both periodic and molecular structures were included in the training set, for each type of structures the details are provided in the following section.

1.1 Periodic systems

Calculations on periodic structures were done with the projector-augmented wave (PAW) method[1,2] as implemented in the Vienna Ab-initio Simulation Package (VASP).[3–5] In line with previous work,[6] most of the reference data was calculated using the PBE exchange-correlation (XC) functional[7] with long-range dispersion interactions accounted for by the DFT-D3(BJ) dispersion correction.[8] The outermost electrons of Br ($4s^2 4p^5$), I ($5s^2 5p^5$), Cs ($5s^2 5p^6 6s^1$) and Pb ($5d^{10} 6s^2 6p^2$) were treated as valence electrons. In the calculations we used a plane-wave cutoff energy of 500 eV together with regularly spaced meshes for the Brillouin zone integration.[9] An overview of the k-meshes used for these calculations can be found in Table S1.

The ground state structures of the precursors and perovskites were obtained by allowing all the ionic positions, cell shape and cell volume to change. During the geometry optimizations, energy and force convergence criteria of 1×10^{-2} meV and 10 meV/Å, respectively, were used. The atomic charges in these materials were calculated with the DDEC6 method.[10,11] Whenever monolayers or slabs were modeled, vacuum layers of at least 15 Å were inserted between the atoms to prevent interactions between the periodic images. Equations of state were generated by applying a strain to the equilibrium geometries in the direction of the lattice vectors and subsequently allowing for the ionic positions to relax using the above-mentioned convergence criteria.

Defect formation energies were calculated for monolayers of PbI_2 and orthorhombic

$CsPbX_3$ (X = Br/I) by taking the difference in energy between the defective and pristine structures. To prevent interactions between the periodic copies of the defects, we used $4 \times 4 \times 1$ and $2 \times 2 \times 1$ supercells for PbI_2 monolayers and orthorhombic $CsPbX_3$, respectively. Accordingly, the k-meshes used to sample reciprocal space were scaled to $3 \times 3 \times 1$ and $2 \times 2 \times 3$. Lower convergence criteria were used to lower the cost of the defect calculations, 1×10^{-1} meV and 30 meV/Å for PbI_2 and 1×10^{-2} meV and 50 meV/Å for $CsPbX_3$.

Defect migration barriers were calculated using transition state calculations with the Climbing Image Nudged Elastic Band (CI-NEB) method.[12,13] For all barriers five intermediate geometries were used for the calculations. The migration barriers in PbI_2 monolayers were calculated in a $4 \times 4 \times 1$ supercell with a $3 \times 3 \times 1$ k-mesh, for computational efficiency the vacuum was reduced to 10 Å here. For $CsPbX_3$ perovskites, we used a $3 \times 3 \times 3$ cubic supercell sampled with only the Γ-point ($1 \times 1 \times 1$ k-mesh). The defect migration barriers in perovskites were calculated using the SCAN XC functional[14] and made use of a Pb pseudopotential with fewer valence electrons ($6s^2 6p^2$).

Table S1: The k-meshes used for the different materials for which an equations of state was calculated. X is used to denote halides (Br or I).

Material	Phase	k-points
$CsPbX_3$	Cubic (α)	$6 \times 6 \times 6$
	Tetragonal (β)	$4 \times 4 \times 6$
	Orthorhombic (γ)	$4 \times 4 \times 3$
	Yellow (δ)	$6 \times 3 \times 2$
CsX	CsCl-type	$12 \times 12 \times 12$
	NaCl-type	$8 \times 8 \times 8$
PbI_2	Hexagonal	$11 \times 11 \times 7$
	MoS_2-type	$11 \times 11 \times 4$
PbI_2 monolayer	Hexagonal monolayer	$11 \times 11 \times 1$
	MoS_2-type monolayer	$11 \times 11 \times 1$
	Octagonal monolayer	$12 \times 12 \times 1$
$PbBr_2$	$PbCl_2$-type	$5 \times 11 \times 5$

1.2 Molecular systems

Molecular geometries PbX_2 (X = Br/I) were evaluated using DFT calculations with ADF[15,16] in AMS2022.[17] For all species (Br, I and Pb) all electrons were treated as valence electrons. The basis set for the calculations was a TZP basis set of Slater Type Orbitals (STO).[18] Scalar relativistic effects were included using the Zero Order Regular Approximated (ZORA) Hamiltonian.[19] Analogous to the majority of the bulk calculations, the PBE XC functional[7] was used to model the electronic exchange-correlation interactions. Dispersion interactions were accounted for by the Becke-Johnson damped DFT-D3 scheme.[8] Consequently, these settings were used to scan the potential energy surface of PbX_2 molecular systems. Using constrained geometry optimizations the energy profile of the $X-Pb-X$ valence angles and $Pb-X$ bonds were scanned.

2 Force field optimization

2.1 Parameter optimization

The parameter optimization was carried out using a covariance matrix adaptation evolutionary strategy (CMA-ES) algorithm proposed by Hansen and Ostermeier [20]. During parameter optimization a set of pre-defined parameters in the ReaxFF force field was allowed to change within set bounds. During each optimization step, a collection of new parameter sets was generated. The quality of the parameter sets in this collection was evaluated using the sum of squared errors (SSE) loss function constructed from the entries in the training set. During successive generations, the distribution of parameter sets is improved upon by moving into the direction of the lowest values for the loss function. We made use of a population size of $N_{\text{pop}} = 16$ for each generation, moreover we used an initial standard deviation for the multivariate normal distribution of $\sigma = 0.2$ from which trial parameter sets could be drawn.

2.2 Parameter scaling

The Br parameters were initialized from scaled atomic and interatomic I parameters as found in the retrained I/Pb/Cs ReaxFF force field. The scaling constants used for the procedure are reported in Table S2. The Br parameters were allowed to change during the parameter optimization procedure. To adhere to the chemical trends for ion size and bond distances, we applied constraints during the parameter optimization. With the constraints, shown in Table S3, we ensured that Van der Waals interactions are longer-ranged than covalent interactions ($r_{\text{vdW}} > r_0^{\text{sigma}}$) and that all I-containing interactions had a longer range than their equivalent Br-containing interactions.

Table S2: Scale constants used to convert I atomic and interatomic parameters to parameters for Br.

Interaction	Parameter	Elements	Scale constants
Bonding (BND)	D_e^{sigma}	Br.I	1.05
		Br.Br	1.10
		Br.Pb	1.10
		Br.Cs	1.10
Off-diagonal (OFD)	D_{ij}	Br.Pb	1.10
		Br.Cs	1.10
	r_{vdW}	Br.Pb	0.95
		Br.Cs	0.95
	r_0^{sigma}	Br.Pb	0.95
		Br.Cs	0.95

Table S3: Scale constants used to convert I atomic and interatomic parameters to parameters for Br.

	Constraint
1.	Cs.I:r_{vdW} > Cs.I:r_0^{sigma}
2.	Br.Cs:r_{vdW} > Br.Cs:r_0^{sigma}
3.	I.Pb:r_{vdW} > I.Pb:r_0^{sigma}
4.	Br.Pb:r_{vdW} > Br.Pb:r_0^{sigma}
5.	Cs.I:r_{vdW} > Br.Cs:r_{vdW}
6.	Cs.I:r_0^{sigma} > Br.Cs:r_0^{sigma}
7.	I.Pb:r_{vdW} > Br.Pb:r_{vdW}
8.	I.Pb:r_0^{sigma} > Br.Pb:r_0^{sigma}

3 Force field validation

3.1 Atomic charges

The electronegativity equalization method (EEM)[21] is used to determine the atomic charges in the ReaxFF force field. For DFT calculations the charges were obtained using the DDEC6 method.[10,11] We note that the new ReaxFF parameters accurately reproduce the atomic charges of different species in a variety of compounds, which include precursor and perovskite geometries. A comparison of the charges from DFT and from ReaxFF is provided in Table S4.

Table S4: Atomic charges of different species in a variety of compounds as compared between DFT and ReaxFF calculations.

Compound	Element	X = I		X = Br	
		q_{DFT} (e)	q_{ReaxFF} (e)	q_{DFT} (e)	q_{ReaxFF} (e)
CsX	Cs	+0.80	+0.72	+0.82	+0.77
	X	−0.80	−0.72	−0.82	−0.77
PbX_2	Pb	+0.55	+0.71	+0.90	+0.88
	X	−0.28	−0.36	−0.45	−0.44
α-$CsPbX_3$	Cs	+0.83	+0.80	+0.84	+0.85
	Pb	+0.81	+0.69	+0.95	+0.73
	X	−0.55	−0.49	−0.60	−0.53
β-$CsPbX_3$	Cs	+0.81	+0.81	+0.83	+0.86
	Pb	+0.80	+0.67	+0.94	+0.73
	X	−0.53	−0.49	−0.58	−0.53
γ-$CsPbX_3$	Cs	+0.78	+0.83	+0.81	+0.87
	Pb	+0.78	+0.67	+0.94	+0.74
	axial X	−0.52	−0.49	−0.58	−0.54
	equatorial X	−0.52	−0.50	−0.58	−0.53

3.2 Equations of state

In addition to the equations of state for $CsPbI_3$ shown in the main text, we show that the ReaxFF force field also captures the bulk phase behavior of $CsPbBr_3$ in Figure S1. We note that the ReaxFF parameter appropriately ranks the various perovskite phases of $CsPbBr_3$ from least to most stable as: $\alpha < \beta < \delta$. The relative energies of the different perovskite and nonperovskite phases are provided in Table S5.

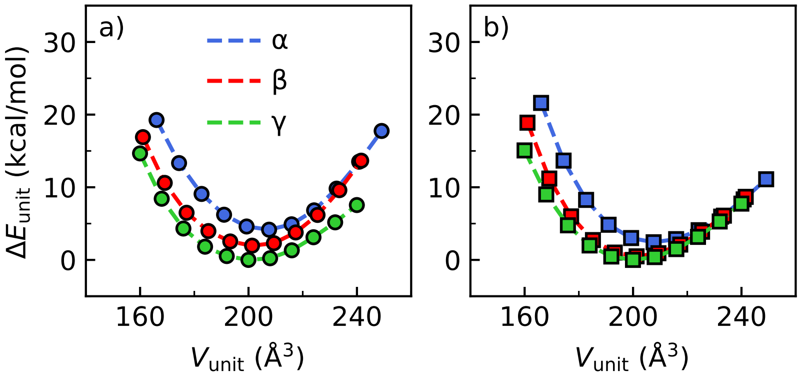

Figure S1: Equations of state of various phases of $CsPbBr_3$ from a) ReaxFF and b) DFT calculations.

Table S5: Relative energies in kcal/mol **of the various phases of $CsPbI_3$ and $CsPbBr_3$ with respect to the γ-phase.**

Phase	$CsPbI_3$		$CsPbBr_3$	
	DFT	ReaxFF	DFT	ReaxFF
α	+3.54	+2.88	+2.43	+4.16
β	+0.96	+1.33	+0.49	+1.96
γ	+0.00	+0.00	+0.00	+0.00
δ	−3.06	−5.87	-	-

3.3 Mixing enthalpies

The mixing enthalpy of $CsPb(Br_xI_{1-x})_3$ perovskites was determined by comparing the total energy of mixed halide perovskites to that of the pure perovskites (i.e. $CsPbI_3$ and $CsPbBr_3$). The following equation was used to calculate the mixing enthalpies

$$E_{\text{unit}}^{\text{mix}} = E_{\text{unit}}^{\text{CsPb(Br}_\text{x}\text{I}_{1-\text{x}})_3} - x \cdot E_{\text{unit}}^{\text{CsPbBr}_3} - (1-x) \cdot E_{\text{unit}}^{\text{CsPbI}_3} \tag{S1}$$

here E_{unit} is the total energies per formula unit for $CsPb(Br_xI_{1-x})_3$, $CsPbBr_3$ and $CsPbI_3$. For each mixed halide perovskite, the lowest energy structure from a set of six randomly mixed orthorhombic structures was used to determine the mixing enthalpy. Due to this sampling, it is possible that an outlier geometry was selected in the determination of the mixing enthalpy (e.g. only axial substitutions for x = 1/6 and x = 1/4), thus resulting in a too low mixing enthalpy. The resulting mixing enthalpies, shown in Table S6, corroborate previous DFT calculations in literature in which mixing enthalpies below 1.0 kcal/mol per formula unit were also found.[22]

Table S6: Mixing enthalpies of $CsPb(Br_xI_{1-x})_3$ perovskites determined with ReaxFF and DFT calculations in kcal/mol**.**

x	x	DFT	ReaxFF
0	0/12	+0.00	+0.00
1/12	1/12	+0.14	+0.15
1/6	2/12	+0.08	−0.39
1/4	3/12	+0.11	−0.10
1/3	4/12	+0.50	+0.53
5/12	5/12	+0.71	+0.83
1/2	6/12	+0.68	+0.64
7/12	7/12	+0.55	+0.51
2/3	8/12	+0.39	+0.56
3/4	9/12	+0.47	+0.37
5/6	10/12	+0.26	−0.04
11/12	11/12	+0.19	−0.03
1	12/12	+0.00	+0.00

3.4 $CsPbI_3$ degradation pathway

To check if the new ReaxFF force field improves on the description of the commonly observed degradation of $CsPbI_3$, we compared the I/Pb/Cs and I/Br/Pb/Cs ReaxFF parameters against the degradation mechanism of $CsPbI_3$ from the γ-phase to the δ-phase as proposed by Chen et al.[23]. This comparison is shown in Figure S2. Notably, we find that the new set of ReaxFF parameters shows important improvements for the various metastable states (MS1, MS2 and MS3) and final state (δ) along the reaction coordinate, which are overstabilized anywhere from 3 kcal/mol to 9 kcal/mol per formula unit with the I/Pb/Cs parameter set. Moreover, we find that the reparameterized ReaxFF parameters provide more reasonable energies for some transition states (TS1 and TS3), the values of which are closer to the reference values from DFT calculations by about 1.0 kcal/mol to 1.5 kcal/mol per formula unit. A complete overview of all relative energies is shown in Table S7.

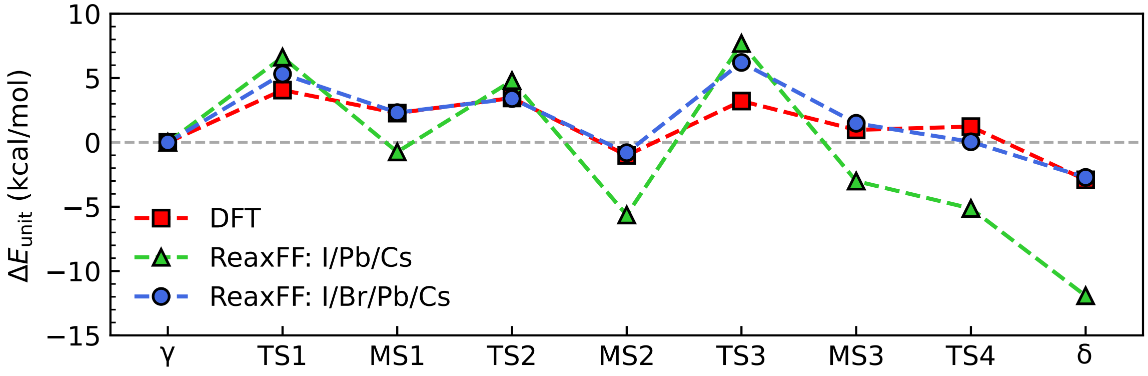

Figure S2: $CsPbI_3$ degradation mechanism from the γ-phase to the δ-phase. Reference data from DFT calculations (squares) are compared to the I/Pb/Cs (triangles) and I/Br/Pb/Cs (circles) ReaxFF parameters.

Table S7: Relative energies per unit cell for DFT, I/Pb/Cs ReaxFF and I/Br/Pb/Cs ReaxFF calculations. Energies are provided in kcal/mol**.**

Phase	DFT	ReaxFF: I/Pb/Cs	ReaxFF: I/Br/Pb/Cs
γ	+0.00	+0.00	+0.00
TS1	+4.07	+6.62	+5.32
MS1	+2.28	−0.74	+2.32
TS2	+3.46	+4.80	+3.41
MS2	−1.08	−5.81	−0.94
TS3	+3.22	+7.69	+6.21
MS3	+0.97	−3.01	+1.49
TS4	+1.23	−5.14	+0.05
δ	−2.91	−11.92	−2.72

3.5 Defect migration barriers

In addition to I vacancies in $CsPbI_3$, we also calculated the migration barrier for other types of defects in inorganic halide perovskites as shown in Figure S3; I interstitial in $CsPbI_3$, Br vacancy in $CsPbBr_3$ and Br interstitial in $CsPbBr_3$. We note that all defect migration barriers match the DFT migration barriers to within 1.5 kcal/mol.

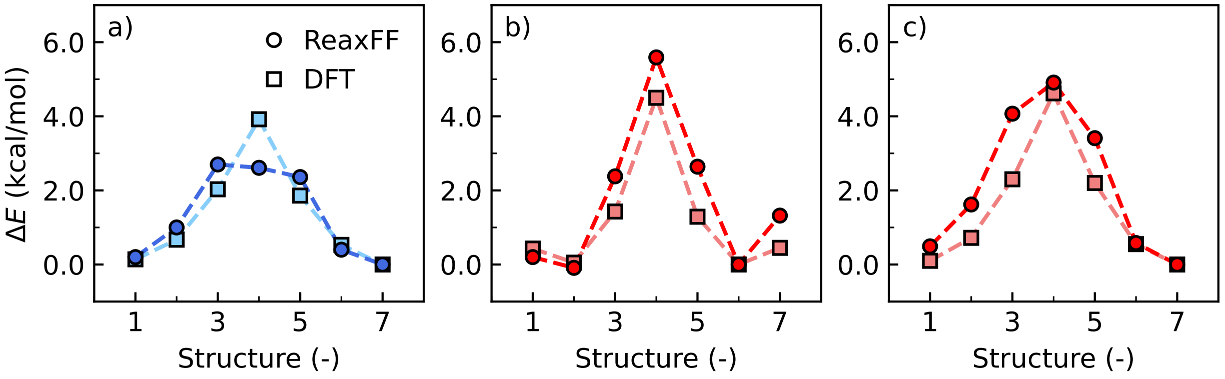

Figure S3: Defect migration barriers of a) I interstitial in $CsPbI_3$, b) Br vacancy in $CsPbBr_3$ and c) Br interstitial in $CsPbBr_3$. Data from the ReaxFF force field are shown in circles and from DFT calculations in squares.

3.6 PbX_2 molecular geometries

To validate that the ReaxFF parameter set works not only for bulk structures, but also molecular fragments, we benchmarked the parameter set against some PbX_2 molecular geometries. For both PbI_2 and $PbBr_2$ we scanned the $Pb - X$ bond distance and $X - Pb - X$ valence angle. The results of these scans can be found in Figure S4 and show a good agreement between ReaxFF and the DFT reference data.

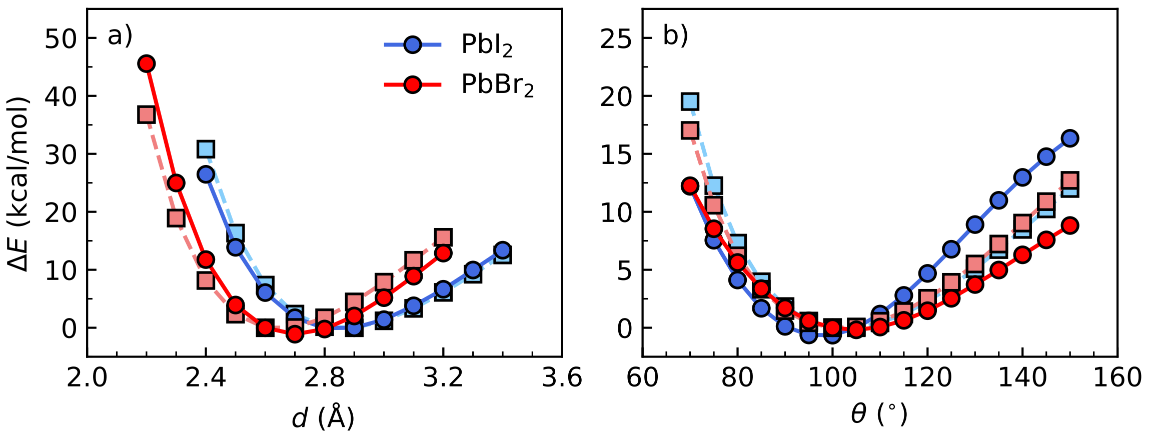

Figure S4: Geometry scans of PbI_2 and $PbBr_2$ molecules. a) $Pb - X$ bond distance scan and b) $X - Pb - X$ valence angle scan. Data from the ReaxFF force field are shown in circles and from DFT calculations in squares.

3.7 PbI_2 precursor geometry

To validate the force field in dynamical situations, we compared the finite temperature geometry of hexagonal (2H) PbI_2 with experiments.[24] We simulated the material at 300 K and atmospheric pressure. First an equilibration run of 50 ps was done, following this equilibration run a production run of 200 ps was started. The lattice vectors (Table S8) were averaged over the full duration of the production runs.

Table S8: Comparison of lattice vectors of PbI_2 from experiments[24] with ReaxFF simulations.

	a (Å)	c (Å)
Experiments	4.56	6.98
ReaxFF (300 K)	4.65	6.85

4 Mixed halide model systems

Using the ^{207}Pb chemical shift to probe the local chemical environment of Pb in inorganic halide perovskites, Karmakar et al.[25] established that halides are randomly distributed in the perovskite lattice. As such, we create mixed halide perovskite systems by randomly placing different halide species (i.e. I or Br) on the X-site of the AMX_3 perovskite lattice. By varying the number of I and Br species, we can control the final composition of the model system. In the creation of the structures, we ensured that an equal number of halides were substituted along each of the principal axes of the PbX_6 octahedra for a homogeneous halide distribution. Examples of cubic $CsPb(Br_xI_{1-x})_3$ mixed halide perovskites are shown in Figure S5.

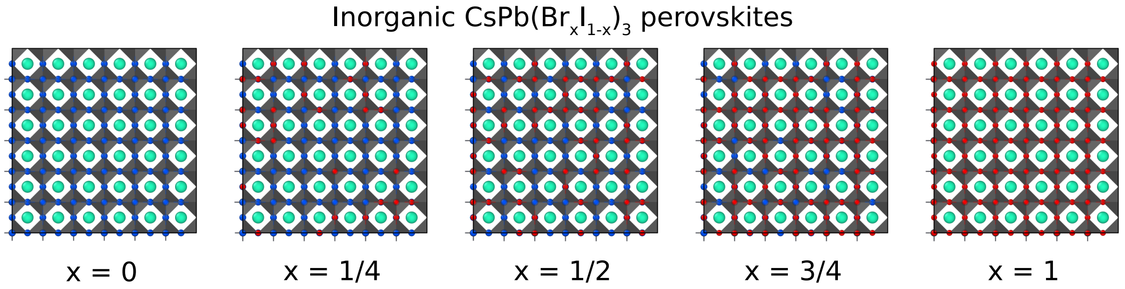

Figure S5: Mixed halide cubic $CsPb(Br_xI_{1-x})_3$ models created through random placement of different halide species on the inorganic sublattice. Iodide and bromide species are colored blue and red, respectively.

5 Molecular dynamics simulations

The ReaxFF molecular dynamics simulations were done in AMS2022,[17] using the $CsPb(Br_xI_{1-x})_3$ force field developed in this work. In all simulations a time step of 0.25 fs was used and the atom positions were written out every 400 steps (0.1 ps). All simulations were carried out in an NpT-ensemble in which the temperature and pressure were controlled using a thermostat and barostat with damping constants of $\tau_T = 100$ fs and $\tau_p = 2500$ fs, respectively. In the simulations, the initial velocities were assigned according to a Maxwell-Boltzmann distribution at the initial temperature. During the equilibration runs we made use of the Berendsen thermostat and barostat[26] to control the pressure and temperature. The information of the final frame (i.e. positions and velocities) was then used as the starting point of the production runs. For the production runs, we employed the Nosé-Hoover chains (NHC) thermostat ($N_{chain} = 10$)[27] and Martyna-Tobias-Klein (MTK) barostat[28] for pressure and temperature control, respectively.

5.1 Unit cell volumes

To investigate the unit cell volumes predicted by ReaxFF simulations, $4 \times 4 \times 3$ supercells of orthorhombic $CsPb(Br_xI_{1-x})_3$ were used. The systems were first equilibrated to the target temperature (300 K) at atmospheric pressure for 50 ps. After these runs, production runs were started to sample the unit cell volume for 200 ps. The volume of the pseudocubic unit cell V_{pc} was obtained by dividing the average of the total system volume by the number of formula units in the supercell. The pseudocubic lattice vector then calculated, under the assumption of cubic unit cells, as $a = \sqrt[3]{V_{pc}}$.

5.2 Phase diagrams

Phase diagrams were created with $4 \times 4 \times 3$ supercells of orthorhombic $CsPb(Br_xI_{1-x})_3$. All compositions were initially equilibrated to 100 K at atmospheric pressure for 25 ps. All systems were then gradually heated from 100 K to 700 K during a 1.2 ns simulation, resulting in a heating rate of 0.5 K/ps. Pseudocubic lattice vectors a, b and c were consequently obtained by converting the orthorhombic cell vectors a_o, b_o and c_o as: $a = a_o/\sqrt{2}$, $b = b_o/\sqrt{2}$ and $c = c_o/2$. A window averaging with a window width of $\Delta t_w = 10.0$ ps was used to smooth the fluctuations in phase diagrams. Figure S6 demonstrates that the smoothing of this data does not impact the location of the phase transitions in $CsPbI_3$. Moreover, sensitivity tests of the phase diagrams for the heating rate (Figure S7), supercell size (Figure S8) and halide mixing (Figure S9) show the robust behavior of the phase diagrams of the inorganic perovskites. We note that hysteresis effects have been observed during the rapid cooling of $CsPbI_3$ before,[6] yet, by focusing on the phase diagrams from the heating of the inorganic perovskites, such hysteresis effects do not have to be taken into account.

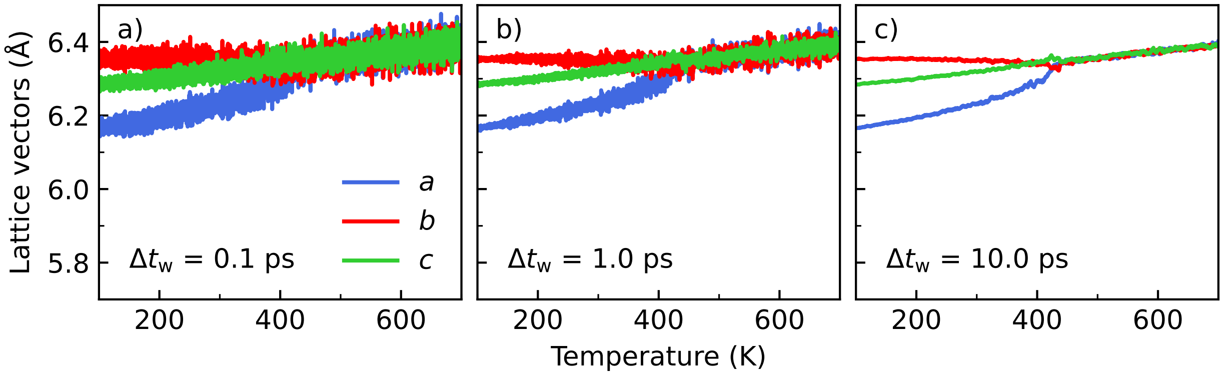

Figure S6: Effects of window averaging on the phase diagram of $CsPbI_3$. The investigated window widths are a) no window (0.1 ps), b) 1.0 ps and c) 10.0 ps.

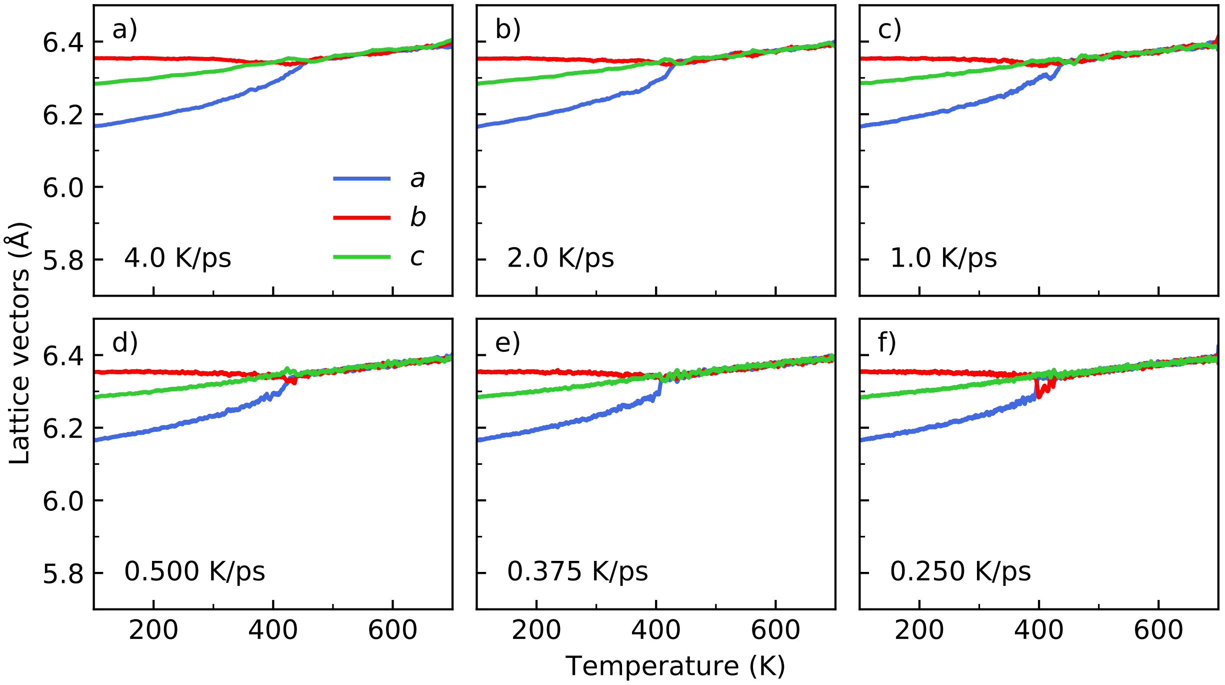

Figure S7: Effects of heating rate on the phase diagram of $CsPbI_3$. The investigated heating rates are a) 4.0 K/ps, b) 2.0 K/ps, c) 1.0 K/ps, d) 0.500 K/ps e) 0.375 K/ps and f) 0.250 K/ps.

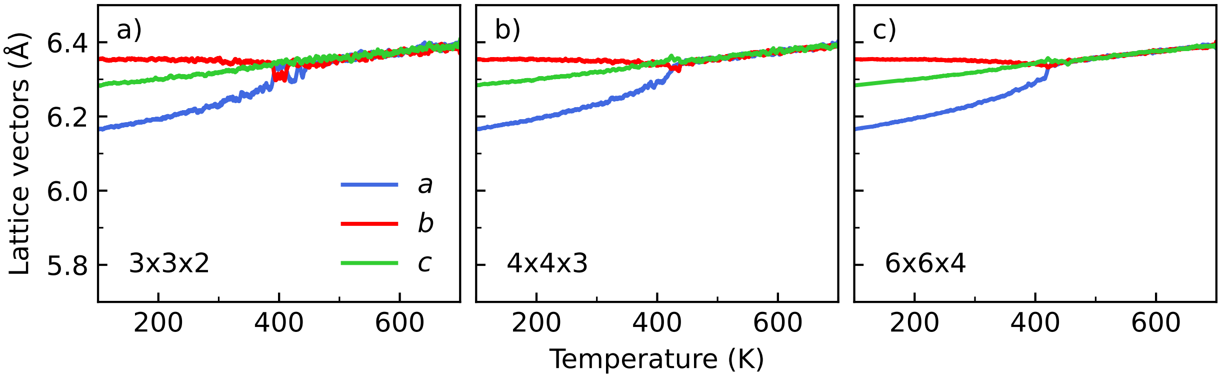

Figure S8: Effects of supercell size on the phase diagram of $CsPbI_3$. The investigated supercell sizes are a) $3 \times 3 \times 2$, b) $4 \times 4 \times 3$ and c) $6 \times 6 \times 4$.

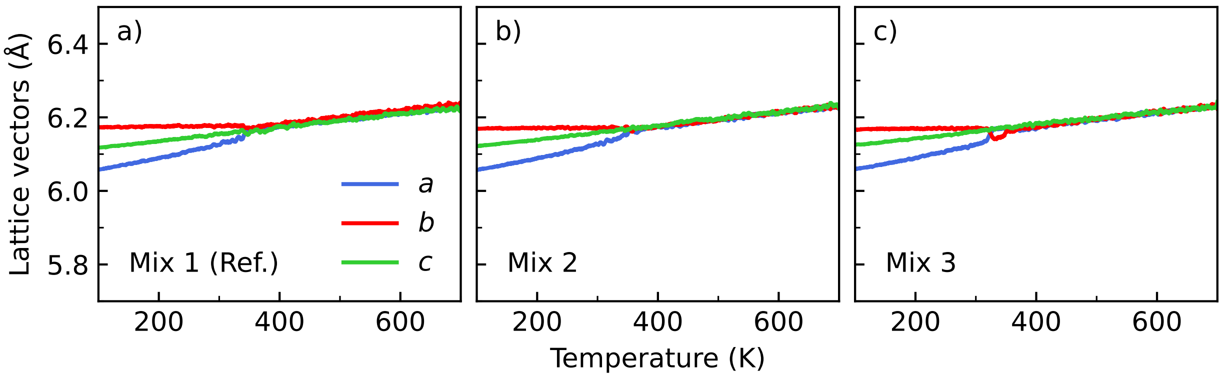

Figure S9: Effects of differences in halide mixing on the phase diagram of $CsPb(Br_{1/4}I_{3/4})_3$. Three model systems with identical halide ratios are compared in the subfigures.

5.3 Homogeneously mixed systems

The octahedral dynamics in homogeneously mixed systems were analyzed in $4 \times 4 \times 3$ orthorhombic supercells. The model systems were equilibrated to 75 K at atmospheric pressure for 50 ps. During the production runs (1.3 ns) the temperature was gradually increased to 725 K, resulting in a heating rate of 0.5 K/ps. To obtain octahedral distributions, we divided the trajectory in sections of 100 ps, each representing a temperature window of 50 K. The distributions were analyzed using the functional forms described in SI Note 6. The temperature evolution of the average octahedral tilt angles was obtained by dividing the trajectory into sections of 20 ps (i.e. 10 K windows). The temperature evolution of θ_x and θ_y can be found in Figure S10 and Figure S11, respectively.

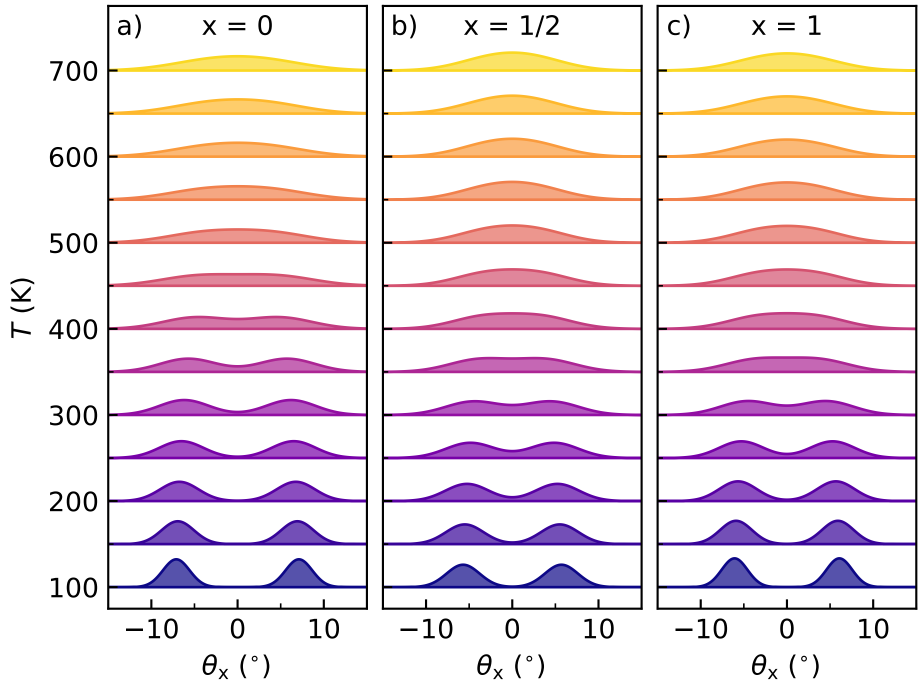

Figure S10: Temperature evolution of the octahedral orientation θ_x for $CsPb(Br_xI_{1-x})_3$ perovskites with compositions a) x = 0, b) x = 1/2 and c) x = 1.

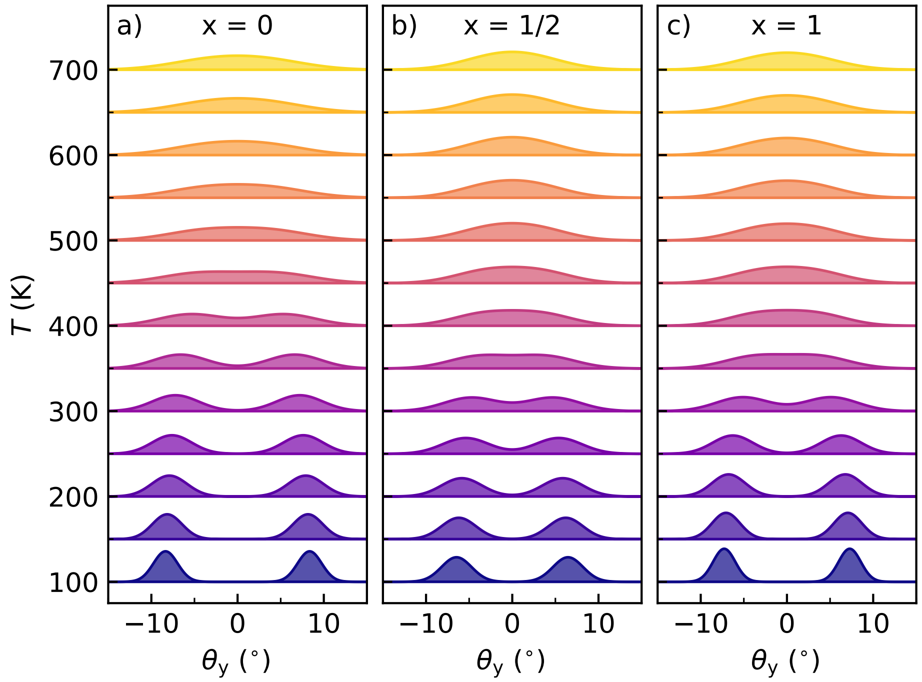

Figure S11: Temperature evolution of the octahedral orientation θ_y for $CsPb(Br_xI_{1-x})_3$ perovskites with compositions a) x = 0, b) x = 1/2 and c) x = 1.

5.4 Dilute mixed systems

To investigate the local effects of halide substitutions in the inorganic perovskites, we used $4 \times 4 \times 3$ orthorhombic supercells. From these cells we randomly selected a Pb species and substituted one or two of the neighboring halides species on either the axial or equatorial sites. The model systems were then equilibrated to the target temperature of 300 K at atmospheric pressure for 50 ps, followed by a production run of 500 ps to sample the octahedral dynamics. The average tilt angles $\langle\theta_\mathrm{i}\rangle$ and standard deviations in those tilt angles σ_i (i = x, y, z) as obtained during the full production runs are shown in Table S9.

Table S9: Averages $\langle\theta_\mathrm{i}\rangle$ and standard deviations σ_i (i = x, y, z) of the octahedral tilting of PbX_6 octahedra in pure $CsPbI_3$, pure $CsPbBr_3$ and substituted $CsPbI_3$ at 300 K**.**

Octahedral type	$\langle\theta_\mathrm{x}\rangle$ (°)	$\langle\theta_\mathrm{y}\rangle$ (°)	$\langle\theta_\mathrm{z}\rangle$ (°)	σ_x (°)	σ_y (°)	σ_z (°)
$CsPbI_3$	6.20	7.22	8.93	2.88	2.66	2.51
Axial substitution	5.67	5.55	8.50	2.34	2.21	2.61
Equatorial substitution	4.80	5.52	7.11	2.76	2.48	2.29
$CsPbBr_3$	4.59	4.57	4.48	3.41	3.39	3.38

6 Octahedral orientation

6.1 Determination of orientation

We determine the orientation of the octahedra in the inorganic PbX_6 framework of the inorganic halide perovskites using polyhedral template matching (PTM) as implemented in `OVITO`.[29,30] The neighborhood of PbX_6 octahedra closely resembles the `Simple Cubic` environment. Using a root-mean-square deviation (RMSD) cutoff for PTM of 0.2, a robust identification of the octahedra is realized in both pure and mixed halide perovskites. The orientation of the octahedra is consequently given in terms of the Euler angles (θ_x, θ_y and θ_z) using the x-y-z order Tait-Bryan convention with extrinsic rotations (see Figure 4a). Whenever the orthorhombic structure of $CsPbX_3$ perovskites is used, we rotate the coordinate axis with respect to which the octahedral orientation is determined with 45° around the z-axis. The rotated coordinate axis is shown in Figure S12.

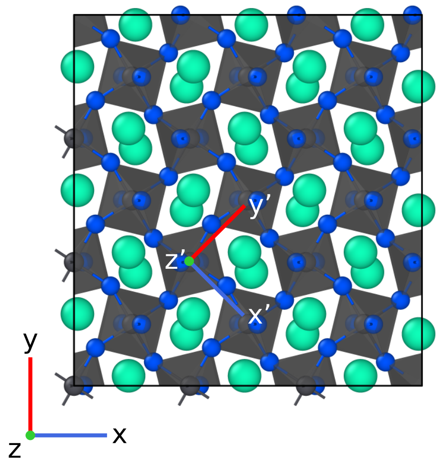

Figure S12: Rotated coordinate axis ($x'y'z'$) used to determine the octahedral orientation for PbI_6 octahedra in orthorhombic $CsPbI_3$. The original coordinate axis (xyz) is also shown.

6.2 Analysis of orientation

The location and spread of the octahedral tilting distributions can consequently be analyzed using Gaussian distributions. Due to an out-of-phase tilting pattern of the octahedra in low-temperature $CsPbX_3$ phases, the octahedral angles have a bimodal tilting distribution. Such bimodal distributions can be analyzed using a symmetric Gaussian function around zero[31] of the form

$$g_2(\theta_\mathrm{i}) = \frac{1}{2\sigma_\mathrm{i}\sqrt{2\pi}} \left(\exp\left[-\frac{(\theta_\mathrm{i} - \langle\theta_\mathrm{i}\rangle)^2}{2\sigma_\mathrm{i}^2} \right] + \exp\left[-\frac{(\theta_\mathrm{i} + \langle\theta_\mathrm{i}\rangle)^2}{2\sigma_\mathrm{i}^2} \right] \right) \tag{S2}$$

where θ_i is an arbitrary octahedral angle (i = x, y, z), $\langle\theta_\mathrm{i}\rangle$ is the average tilting angle and σ_i is the standard deviation in the tilting angle. Whenever the tilting distribution of a single PbX_6 octahedron is investigated, a single Gaussian function is used

$$g_1(\theta_\mathrm{i}) = \frac{1}{\sigma_\mathrm{i}\sqrt{2\pi}} \exp\left[-\frac{(\theta_\mathrm{i} - \langle\theta_\mathrm{i}\rangle)^2}{2\sigma_\mathrm{i}^2} \right]. \tag{S3}$$

To validate the use of the aforementioned Gaussian distributions for analysis of the octahedral distributions, we compare fits of g_1 and g_2 to octahedral distributions from simulations. Figure S13 shows that the Gaussian distributions match well with the distributions obtained from ReaxFF simulations at 300 K. Specifically, we find that the double Gaussian distribution describes the collective orientation of PbI_6 octahedra in pure $CsPbI_3$ (Figure S13a). Moreover, the tilting distributions of both individual pure and substituted octahedra in inorganic perovskites (Figure S13b and Figure S13c) are well-described by a single Gaussian distribution.

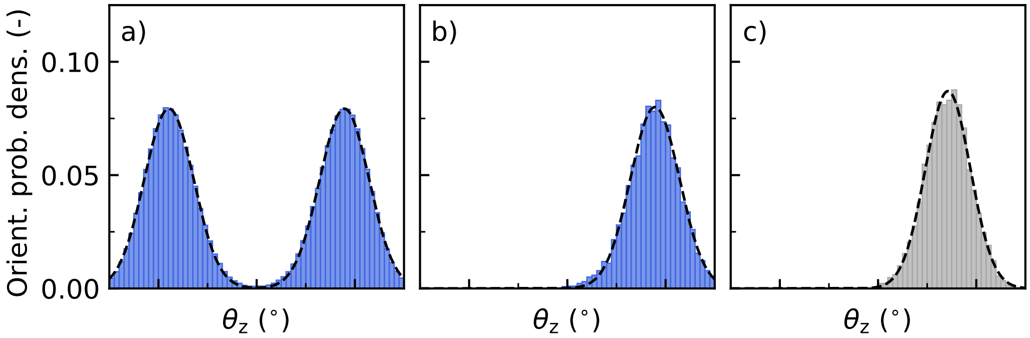

Figure S13: Comparison of octahedral tilting distributions of θ_z with fits with Gaussian distributions. Tilting distributions of a) PbI_6 octahedra in pure $CsPbI_3$, b) a single PbI_6 octahedron in $CsPbI_3$ and c) a single octahedron in $CsPbI_3$ that is doubly substituted by Br in the equatorial direction. In all plots, the dashed black line shows the Gaussian fits, whereas the simulated distributions are depicted with colored bars.

7 Substitution propagation

7.1 Single substitution

To highlight that the substitution of a single halide is enough to impact the dynamics of neighboring octahedra, we assess the octahedral dynamics in $CsPbI_3$ with a single Br substitution in the equatorial direction in Figure S14. We find a similar strain effect for such single halide substitutions as for double halide substitutions. Interestingly, the effect appears to be slightly smaller for single substitutions than for double substitutions as shown in Table S10, hinting at an additive nature of this strain effect.

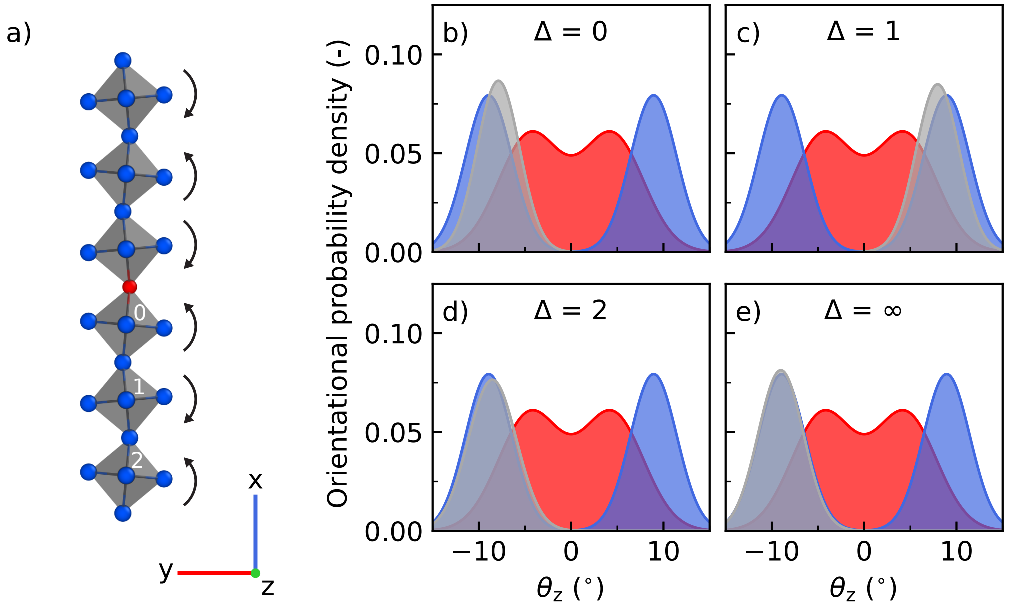

Figure S14: a) Chain of PbX_6 octahedra with a single substitution. The numbers in the octahedra indicate the distance relative to the substituted octahedron. Distribution of θ_z of b) substituted octahedron ($\Delta = 0$), c) direct neighbor of the substituted octahedron ($\Delta = 1$), d) octahedron two sites away from the substituted octahedron ($\Delta = 2$) and e) reference octahedron very far away from the halide substitution ($\Delta = \infty$). The tilting distributions of the investigated octahedra are shown in gray, and those for $CsPbI_3$ and $CsPbBr_3$ in blue and red, respectively.

Table S10: Averages $\langle\theta_z\rangle$ and standard deviations σ_z in the octahedral tilting of PbX_6 octahedra in equatorially substituted $CsPbI_3$ at 300 K in the dilute limit. The distance from the substituted octahedron is expressed in Δ.

Δ	2 substitutions		1 substitution	
	$\langle\theta_z\rangle$ (°)	σ_z (°)	$\langle\theta_z\rangle$ (°)	σ_z (°)
0	7.11	2.29	7.88	2.30
1	7.50	2.46	7.96	2.35
2	7.70	2.44	8.54	2.61
∞	9.01	2.49	9.02	2.45

7.2 Propagation distance

To determine the range of the strain effect of halide substitutions, we assess the octahedral orientations around a double equatorial halide substitution in a larger orthorhombic supercell ($6 \times 6 \times 4$). The resulting octahedral distributions are shown in Figure S15, where we used the same naming convention as in Figure 6, with Δ indicating the distance of the octahedra away from the substitution. The overview of the tilt angles (Table S11) demonstrates that the propagation of the strain effect diminishes significantly for octahedra spaced further than three sites away from the substitution ($\Delta > 3$), indicating a propagation distance of 2 nm for this strain effect in the direction of the substitutions.

Table S11: Averages $\langle\theta_z\rangle$ and standard deviations σ_z in the octahedral tilting of PbX_6 octahedra in $6 \times 6 \times 4$ supercells of $CsPbI_3$ at 300 K. The distance from the equatorially substituted octahedron is expressed in Δ.

Δ	$\langle\theta_z\rangle$ (°)	σ_z (°)
0	7.28	2.26
1	7.60	2.41
2	7.75	2.45
3	8.46	2.39
4	8.59	2.54
5	8.78	2.52
∞	8.82	2.53

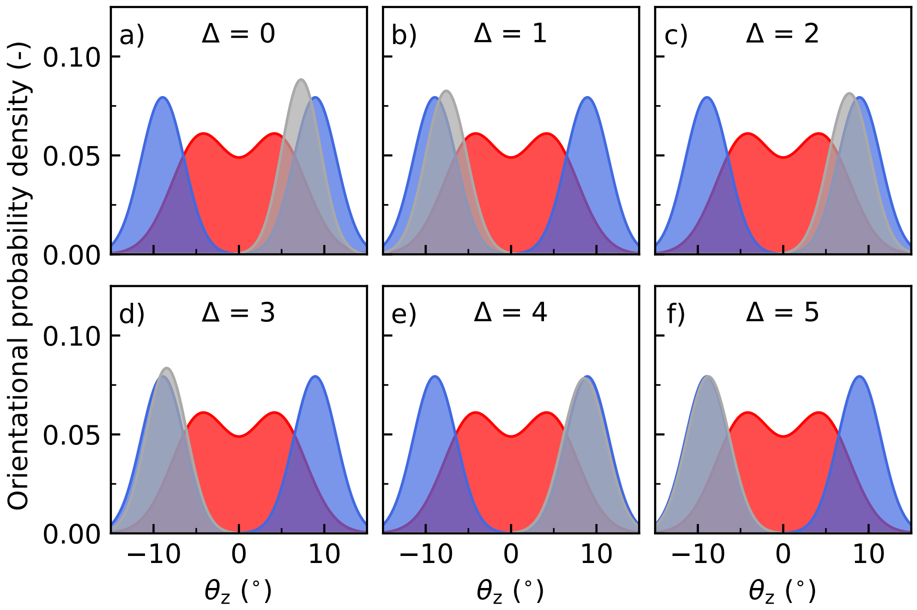

Figure S15: Octahedral distributions of θ_z for the a) substituted octahedron ($\Delta = 0$) and octahedra b) one ($\Delta = 1$), c) two ($\Delta = 2$), d) three ($\Delta = 3$), e) four ($\Delta = 4$) and f) five ($\Delta = 5$) octahedra away from the substituted octahedron as indicated by Δ.

7.3 Perpendicular propagation

To illustrate that halide substitutions do not solely affect octahedra in the direction of the substitution, we show the changes in the octahedral tilting for octahedra arranged perpendicular from the substitution direction in Figure S16. Although the effect is significantly smaller for such octahedra, it results in shifts of about 0.6° for the direct neighbors as shown in Table S12. As is demonstrated by the negligible impact on octahedra spaced two sites away from the substitution, this effect has a considerably shorter range in direction perpendicular to the substitution ($< 1.0\,\mathrm{nm}$) than in the direction of the substitution ($2\,\mathrm{nm}$).

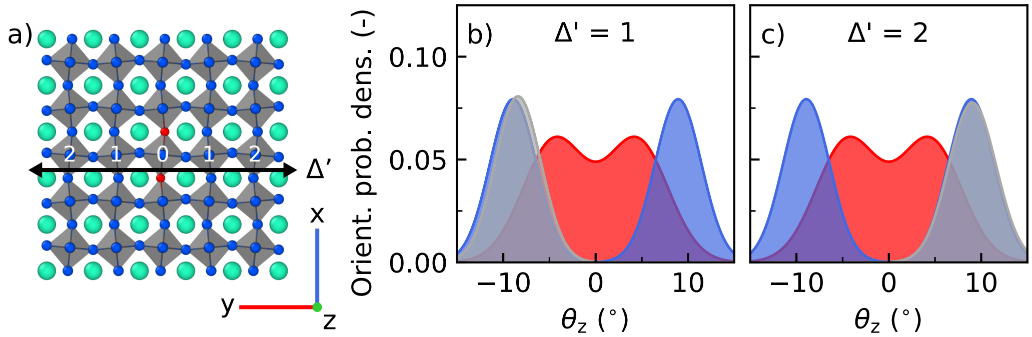

Figure S16: a) Schematic overview of the halide substitution and the investigated octahedra. The numbers in the octahedra indicate the distance relative to the substituted octahedron. Distribution of θ_z of b) direct neighbor of the substituted octahedron ($\Delta' = 1$) and c) octahedron two sites away from the substituted octahedron ($\Delta' = 2$). The tilting distributions of the investigated octahedra are shown in gray, and those for $CsPbI_3$ and $CsPbBr_3$ in blue and red, respectively.

Table S12: Averages $\langle\theta_z\rangle$ and standard deviations σ_z in the octahedral tilting of PbX_6 octahedra in substituted $CsPbI_3$ at 300 K in the dilute limit. The perpendicular distance from the substituted octahedron is expressed in Δ'. The tilting distributions of the investigated octahedra are shown in gray, and those for $CsPbI_3$ and $CsPbBr_3$ in blue and red, respectively.

Δ'	$\langle\theta_z\rangle$ (°)	σ_z (°)
0	7.11	2.29
1	8.40	2.47
2	9.05	2.57
∞	9.01	2.49

8 Tolerance factors

The Goldschmidt tolerance factor is an empirical factor to predict the distortion of an AMX_3 perovskite crystal structure[32] as

$$t = \frac{r_A + r_X}{\sqrt{2}\,(r_M + r_X)} \tag{S4}$$

where r_A, r_M and r_X are the ionic radii of A, M and X ions, respectively. Materials with a tolerance factor of $0.90-1.00$ have a cubic perovskite structure, lower tolerance factors ($0.71-0.90$) indicate an orthorhombic perovskite structure with tilted octahedra and materials with very high (> 1.00) or very low (< 0.71) tolerance factors suggest the formation of nonperovskite structures.

Using the ionic radii from Shannon[33] shown in Table S13, the Goldschmidt tolerance factors of $CsPbX_3$ perovskites can be calculated using equation S4. $CsPbBr_3$ (0.815) has a slightly higher tolerance factor than $CsPbI_3$ (0.807), indicating it has a slightly less distorted perovskite structure.

Table S13: Ionic radii reported by Shannon[33].

Element	r_{ion} (Å)
Cs	1.67
Pb	1.19
Br	1.96
I	2.20

References

(1) Blöchl, P. E. Projector Augmented-Wave Method. *Phys. Rev. B* **1994**, *50*, 17953–17979.

(2) Kresse, G.; Joubert, D. From Ultrasoft Pseudopotentials to the Projector Augmented-Wave Method. *Phys. Rev. B* **1999**, *59*, 1758–1775.

(3) Kresse, G.; Hafner, J. Ab Initio Molecular-Dynamics Simulation of the Liquid-Metal–Amorphous-Semiconductor Transition in Germanium. *Phys. Rev. B* **1994**, *49*, 14251–14269.

(4) Kresse, G.; Furthmüller, J. Efficiency of Ab-Initio Total Energy Calculations for Metals and Semiconductors Using a Plane-Wave Basis Set. *Comput. Mater. Sci.* **1996**, *6*, 15–50.

(5) Kresse, G.; Furthmüller, J. Efficient Iterative Schemes for Ab Initio Total-Energy Calculations Using a Plane-Wave Basis Set. *Phys. Rev. B* **1996**, *54*, 11169–11186.

(6) Pols, M.; Vicent-Luna, J. M.; Filot, I.; van Duin, A. C. T.; Tao, S. Atomistic Insights Into the Degradation of Inorganic Halide Perovskite $CsPbI_3$: A Reactive Force Field Molecular Dynamics Study. *J. Phys. Chem. Lett.* **2021**, *12*, 5519–5525.

(7) Perdew, J. P.; Burke, K.; Ernzerhof, M. Generalized Gradient Approximation Made Simple. *Phys. Rev. Lett.* **1996**, *77*, 3865–3868.

(8) Grimme, S.; Ehrlich, S.; Goerigk, L. Effect of the Damping Function in Dispersion Corrected Density Functional Theory. *J. Comput. Chem.* **2011**, *32*, 1456–1465.

(9) Monkhorst, H. J.; Pack, J. D. Special Points for Brillouin-zone Integrations. *Phys. Rev. B* **1976**, *13*, 5188–5192.

(10) Manz, T. A.; Limas, N. G. Introducing DDEC6 Atomic Population Analysis: Part 1. Charge Partitioning Theory and Methodology. *RSC Adv.* **2016**, *6*, 47771–47801.

(11) Manz, T. A. Introducing DDEC6 Atomic Population Analysis: Part 3. Comprehensive Method to Compute Bond Orders. *RSC Adv.* **2017**, *7*, 45552–45581.

(12) Henkelman, G.; Jónsson, H. Improved Tangent Estimate in the Nudged Elastic Band Method for Finding Minimum Energy Paths and Saddle Points. *J. Chem. Phys.* **2000**, *113*, 9978–9985.

(13) Henkelman, G.; Uberuaga, B. P.; Jónsson, H. A Climbing Image Nudged Elastic Band Method for Finding Saddle Points and Minimum Energy Paths. *J. Chem. Phys.* **2000**, *113*, 9901–9904.

(14) Sun, J.; Ruzsinszky, A.; Perdew, J. P. Strongly Constrained and Appropriately Normed Semilocal Density Functional. *Phys. Rev. Lett.* **2015**, *115*, 036402.

(15) Fonseca Guerra, C.; Snijders, J. G.; te Velde, G.; Baerends, E. J. Towards an Order-N DFT Method. *Theor. Chem. Acc.* **1998**, *99*, 391–403.

(16) te Velde, G.; Bickelhaupt, F. M.; Baerends, E. J.; Fonseca Guerra, C.; van Gisbergen, S. J. A.; Snijders, J. G.; Ziegler, T. Chemistry with ADF. *J. Comput. Chem.* **2001**, *22*, 931–967.

(17) Rüger, R.; Franchini, M.; Trnka, T.; Yakovlev, A.; Philipsen, P.; van Vuren, T., Klumpers, B., Soini, T., *AMS 2022*, SCM, Theoretical Chemistry, Vrije Universiteit, Amsterdam, The Netherlands, 2022.

(18) van Lenthe, E.; Baerends, E. J. Optimized Slater-type Basis Sets for the Elements 1–118. *J. Comput. Chem.* **2003**, *24*, 1142–1156.

(19) van Lenthe, E.; Baerends, E. J.; Snijders, J. G. Relativistic Regular Two-component Hamiltonians. *J. Chem. Phys.* **1993**, *99*, 4597–4610.

(20) Hansen, N.; Ostermeier, A. Completely Derandomized Self-Adaptation in Evolution Strategies. *Evol. Comput.* **2001**, *9*, 159–195.

(21) Mortier, W. J.; Ghosh, S. K.; Shankar, S. Electronegativity-Equalization Method for the Calculation of Atomic Charges in Molecules. *J. Am. Chem. Soc.* **1986**, *108*, 4315–4320.

(22) Chen, Z.; Brocks, G.; Tao, S.; Bobbert, P. A. Unified Theory for Light-Induced Halide Segregation in Mixed Halide Perovskites. *Nat. Commun.* **2021**, *12*, 2687.

(23) Chen, G.-Y.; Guo, Z.-D.; Gong, X.-G.; Yin, W.-J. Kinetic Pathway of γ-to-δ Phase Transition in $CsPbI_3$. *Chem* **2022**, *8*, 3120–3129.

(24) Schlüter, I. Ch.; Schlüter, M. Electronic Structure and Optical Properties of PbI_2. *Phys. Rev. B* **1974**, *9*, 1652–1663.

(25) Karmakar, A.; Dodd, M. S.; Zhang, X.; Oakley, M. S.; Klobukowski, M.; Michaelis, V. K. Mechanochemical Synthesis of 0D and 3D Cesium Lead Mixed Halide Perovskites. *Chem. Commun.* **2019**, *55*, 5079–5082.

(26) Berendsen, H. J. C.; Postma, J. P. M.; van Gunsteren, W. F.; DiNola, A.; Haak, J. R. Molecular Dynamics with Coupling to an External Bath. *J. Chem. Phys.* **1984**, *81*, 3684–3690.

(27) Martyna, G. J.; Klein, M. L.; Tuckerman, M. Nosé–Hoover Chains: The Canonical Ensemble via Continuous Dynamics. *J. Chem. Phys.* **1992**, *97*, 2635–2643.

(28) Martyna, G. J.; Tobias, D. J.; Klein, M. L. Constant Pressure Molecular Dynamics Algorithms. *J. Chem. Phys.* **1994**, *101*, 4177–4189.

(29) Larsen, P. M.; Schmidt, S.; Schiøtz, J. Robust Structural Identification via Polyhedral Template Matching. *Model. Simul. Mater. Sci. Eng.* **2016**, *24*, 055007.

(30) Stukowski, A. Visualization and Analysis of Atomistic Simulation Data with OVITO-The Open Visualization Tool. *Model. Simul. Mater. Sci. Eng.* **2009**, *18*, 015012.

(31) Wiktor, J.; Fransson, E.; Kubicki, D.; Erhart, P. Quantifying Dynamic Tilting in Halide Perovskites: Chemical Trends and Local Correlations. *Chem. Mater.* **2023**, *35*, 6737–6744.

(32) Goldschmidt, V. M. Die Gesetze der Krystallochemie. *Naturwissenschaften* **1926**, *14*, 477–485.

(33) Shannon, R. D. Revised Effective Ionic Radii and Systematic Studies of Interatomic Distances in Halides and Chalcogenides. *Acta Crystallogr. A.* **1976**, *32*, 751–767.
